# Optical and rogue type soliton solutions of the (2+1) dimensional nonlinear Heisenberg ferromagnetic spin chains equation

**DOI:** 10.1038/s41598-023-36536-z

**Published:** 2023-06-19

**Authors:** Shariful Islam, Bishnupada Halder, Ahmed Refaie Ali

**Affiliations:** 1 Department of Electrical and Electronic Engineering, Pundra University of Science and Technology, Bogura, 5800 Bangladesh; 2 Department of Mathematics, Pabna University of Science and Technology, Pabna, 6600 Bangladesh; 3 Department of Mathematics and Computer Science, Faculty of Science, Menoufia University, Shebin El Kom, 32511 Menofia Governorate Egypt

**Keywords:** Engineering, Mathematics and computing, Optics and photonics

## Abstract

In this study, the uses of unified method for finding solutions of a nonlinear Schrödinger equation that describes the nonlinear spin dynamics of (2+1) dimensional Heisenberg ferromagnetic spin chains equation. We successfully construct solutions to these equations. For each of the derived solutions, we provide the parametric requirements for the existence of a valid soliton. In order to visualize some of the discovered solutions, we plot the 2D and 3D graphics. The results of this investigation, which have been presented, might be useful in elucidating the model's physical significance. These are a highly useful tool for studying how electrical solitons, which travel as voltage waves in nonlinear dispersive media, spread out, as well as for doing various physical calculations. The study’s findings, which have been disclosed, might be useful in illuminating the models under consideration's physical significance and electrical field.

## Introduction

PDEs and modern chaos theory concepts have been in close communication thanks to the study of the asymptotic behavior of solutions of nonlinear evolution equations, notably those regulating fluid flows and gas dynamics. This is one of the potential solutions to the central issue of turbulence, one of the most important unsolved issues in the physical sciences. PDEs are crucial in a wide variety of other current mathematical study fields. These include mathematical physics, homogeneous spaces, constructive quantum field theory, infinite dimensional group representations, and so forth.

The cubic nonlinear Schrödinger (NLS) equation

1 $$iu_{t} + u_{xx} + {\text{|u|}}^{{2}} u = 0.$$

has been frequently utilized to simulate the spread of beats in a wide range of physics subfields, such as nonlinear optics^1,2^, water waves^3^, Bose–Einstein condensates^4^ and plasma physics^5^. This model's description of nonlinear wave dynamics has drawn particular interest due to its intriguing accurate solutions of soliton-type envelope. It should be noted that the soliton solutions for the homogeneous NLS equation were first obtained using the inverse scattering transform^4^. A general nonlinear optics model for understanding how light pulses behave in Kerr nonlinear media is the NLS equation. The NLS equation admits bright and dark soliton-type pulse propagation in anomalous and normal dispersion regimes, respectively, for picosecond light pulses and only contains the group velocity dispersion (GVD) and the self-phase modulation, which are well-known in the fiber^6^. The NLS equation also exhibits a variety of nonlinearities, including dual-power laws of nonlinearity, Kerr nonlinearity, cubic nonlinearity, and logarithmic nonlinearity, which has waves with a Gaussian shape that are isolated.

These equations accurately represent real characteristics in nonlinear optics, water waves^1^, Bose–Einstein condensates^2^ and plasma physics^3^ research on NLS equations is booming^3^. The NLS equation has undergone substantial research recently, and it has a variety of applications in optics among other scientific domains. For example, consider the generalized NLS equation with cubic quantity nonlinearities^7^, the RKL model (Radhakrishnan, Kundu and Lakshmanan)^8^, the forced NLS equation with arbitrary nonlinearity^9^, the higher order NLS equation with non-Kerrterms^10^ and the NLS equation with spatiotemporal perturbations^11^. In contrast to the nonlinear Schrödinger equation (NLSE), which governs nonlinear spin dynamics of (2+1)-dimensional Heisenberg ferromagnetic spin chains, it was discovered that the nonlinear spin dynamics of these models with non-Kerr effects are governed by a new integrable NLS type equation in (2+1) dimension sprint. The ability of nonlinear evolution equations to represent nonlinear phenomena in fluid dynamics, nonlinear optics, Bose–Einstein condensates, biological molecules, chemical systems, etc. is well known. The NLSE has conducted extensive research on nonlinear wave propagation in optimism, and in particular fiber optics. The NLSE describes^12,13^ how an envelope function changes over the course of an optical cycle, with the assumption that these changes are gradual.

Heisenberg ferromagnetic models, on the other hand, are crucial to the development of contemporary magnetism theory. The dynamics of magnets that are nonlinear are described. Certain Heisenberg models are provided by partial differential equations that provide soliton solutions and are referred to as soliton equations. To explain the magnetic ordering in ferromagnetic materials, spin–spin interaction models of the sort developed by Heisenberg have been put forth. One of the intriguing groups of nonlinear excitations that describe spin dynamics in semi-classical continuum Heisenberg systems is the magnetic soliton. Analyzing the nonlinear magnetic material properties transform approach^12–14^ can benefit from research into soliton propagation and interaction.

## Application

Application of the Heisenberg ferromagnetic spin chains equation's nonlinear spin dynamics in the (2+1) dimension has been introduced**.** In several domains of nonlinear sciences, including optical fibers, fluid dynamics, plasma physics, and others, nonlinear Schrödinger equations (NLSEs) are frequently employed to describe intricate details^15,16^. Because of the stability between dispersion and nonlinearity effects, Optical solitons are tiny electromagnetic pulses that propagate in nonlinear divergent medium and maintain their strength^17^. The wave-guide can function as a TL, allowing EM to propagate through it and giving the appearance of an optical fibre^18^. Optical solitons have been heralded as the next advancement in high-throughput optical communication technology because of its capacity to transmit signals over long distances without chromatic dispersion^19^. Various soliton solutions have been investigated in various types of NLSEs using a variety of integration strategies. In order to investigate a nonlinear Schrödinger problem under anti cubic, Afzal et al.^20^ used the extended direct algebraic technique. Using the trial solution method, Eslami^21^ investigated the (1+2)-dimensional chiral nonlinear Schrödinger's problem. According to Kumar et al.^21^, the generalized (3+1)-dimensional cubic-quintal nonlinear Schrödinger equation with spatially scattered coefficients has exact spatiotemporal periodic travelling wave solutions. The generalized (3+1)-dimensional nonlinear Schrödinger equation's analytical light bullet solutions were discovered by Belic et al.^22^. In the nonlinear Schrödinger equation with fourth-order dispersion and dual power law nonlinearity, Zayed et al.^23^ studied the soliton solutions. Generally speaking, a number of mathematical techniques have been developed in this field^24–29^.

## The methodology of unified method

The unified approach to solving nonlinear partial differential equations (NPDEs) was described by Gozukizil et al. in their study. Assume that a nonlinear partial differential equation (NPDE), say in two independent variables $x$ and $t$, is given by

2 $$R(f,f_{x} ,f_{xxt} ,f_{xxx} ,f_{t} ,f_{tt} , \ldots ) = 0.$$

where $f(x,t)$ is an unknown function. $R$ is a polynomial in $f = f(x,t)$ and its various derivatives in which the highest order derivatives and non-linear term are both involve.

In this section, we review the crucial idea of the unified method step by step to obtain exact traveling wave solution of NLPDEs.

*Step-01*: To convert the nonlinear partial differential equation into Ordinary differential equation (ODE), we use traveling wave variable

3 $$f(x,t) = f(\xi ),\;\xi = kx - \omega t.$$

where $k$ is constant and $\omega$ is the general termed of the wave velocity. Now substituting Eq. (3) into Eq. (2) we obtain an ordinary differential equation.

4 $$R(f_{\xi } ,f_{\xi \xi \xi } ,f_{\xi \xi } ,ff_{\xi } f_{\xi \xi \xi } , \ldots ) = 0.$$

*Step-02*: Now we integrate Eq. (4) as many times as possible. Keep the integrating constant to zero.

Suppose the solution of ODE Eq. (4) is the following form

5 $$f\left( \xi \right) = l_{0} + \sum\limits_{i = 1}^{N} {\left[ {l_{i} g\left( \xi \right)^{i} + m_{i} g\left( \xi \right)^{ - i} } \right]} .$$

where $$l_{i}$$ $$(i = 0,1,2, \ldots ,N)$$ and $$m_{i}$$$$(i = 0,1,2, \ldots ,N)$$ are constants to be investigated afterward such that $$a_{N}$$ and $$b_{N}$$ cannot be zero at a time. Let us consider an ODE namely Riccati differential equation

6 $$T = \left( {g\left( \xi \right)} \right)^{2} + \lambda .$$

It is satisfied by $f(\xi ).$ The solution of the considering Riccati differential equation are given below.

*Case-01*: Hyperbolic function (when $\lambda < 0$):

$$g\left( \xi \right) = \left\{ \begin{gathered} \frac{{\sqrt { - \left( {p^{2} + r^{2} } \right)\lambda } - p\sqrt { - \lambda } \cosh \left( {2\sqrt { - \lambda } \left( {\xi + q} \right)} \right)}}{{p\sinh \left( {2\sqrt { - \lambda } \left( {\xi + q} \right)} \right) + r}} \hfill \\ \frac{{ - \sqrt { - \left( {p^{2} + r^{2} } \right)\lambda } - l\sqrt { - \lambda } \cosh \left( {2\sqrt { - \lambda } \left( {\xi + q} \right)} \right)}}{{p\sinh \left( {2\sqrt { - \lambda } \left( {\xi + q} \right)} \right) + r}} \hfill \\ \sqrt { - \lambda } + \frac{{2p\sqrt { - \lambda } }}{{p + \cosh \left( {2\sqrt { - \lambda } \left( {\xi + q} \right)} \right) - \sinh \left( {2\sqrt { - \lambda } \left( {\xi + q} \right)} \right)}} \hfill \\ - \sqrt { - \lambda } + \frac{{2p\sqrt { - \lambda } }}{{p + \cosh \left( {2\sqrt { - \lambda } \left( {\xi + q} \right)} \right) - \sinh \left( {2\sqrt { - \lambda } \left( {\xi + q} \right)} \right)}} \hfill \\ \end{gathered} \right.$$

*Case-02*: Trigonometric function (when $\lambda > 0$):

$$g\left( \xi \right) = \left\{ \begin{gathered} \frac{{\sqrt {\left( {p^{2} - r^{2} } \right)\lambda } - p\sqrt \lambda \cos \left( {2\sqrt \lambda \left( {\xi + q} \right)} \right)}}{{p\sin \left( {2\sqrt \lambda \left( {\xi + q} \right)} \right) + r}} \hfill \\ \frac{{ - \sqrt {\left( {p^{2} - r^{2} } \right)\lambda } - p\sqrt \lambda \cos \left( {2\sqrt \lambda \left( {\xi + q} \right)} \right)}}{{p\sin \left( {2\sqrt \lambda \left( {\xi + q} \right)} \right) + r}} \hfill \\ i\sqrt \lambda - \frac{2pi\sqrt \lambda }{{p + \cos \left( {2\sqrt \lambda \left( {\xi + q} \right)} \right) - i\sin \left( {2\sqrt \lambda \left( {\xi + q} \right)} \right)}} \hfill \\ - i\sqrt \lambda + \frac{2li\sqrt \lambda }{{p + \cos \left( {2\sqrt \lambda \left( {\xi + q} \right)} \right) + i\sin \left( {2\sqrt \lambda \left( {\xi + q} \right)} \right)}} \hfill \\ \end{gathered} \right.$$

where $l \ne 0$ and $d$ $g\left( \xi \right)$ are real arbitrary constant.

*Case-03*: Rational function solutions (when $\lambda = 0.$): $g\left( \xi \right) = \frac{1}{\xi + q}.$

*Step-03*: We determine the positive integer $N$ in Eq. (4) by taking into account the homogeneous balance between the highest order derivatives and the nonlinear terms in Eq. (4). Moreover the degree of $U$ as $D(U(\xi )) = N$ which gives the order of others expression as follows:

7 $$D\left( {\frac{{d^{q} U}}{{d\xi^{q} }}} \right) = N + q,\;D\left( {U^{p} \left( {\frac{{d^{q} U}}{{d\xi^{q} }}} \right)^{s}} \right) = sN + s(N + q).$$

Therefore we can obtain the value of $N$ in Eq. (5) with the help of above formula.

*Step-04*: Inserting Eq. (5) into Eq. (4) and making use of Eq. (6) and then extracting all terms of like powers of $$S\left( \xi \right)$$ together, then set each coefficient of them to zero yield a over-determined system of algebraic equations and then solving this system of algebraic equations $$a_{i}$$$$(i = 0,1,2, \ldots ,N)$$ and $$b_{i}$$$$(i = 0,1,2, \ldots ,N)$$ $$k,\omega$$ we obtain several sets of solutions.

*Step-05*: Substituting $$a_{i} ,$$$$b_{i} ,$$$$k$$ and $$\omega$$ into Eq. (5) which is obtained in Step 4 and using the general solutions of Eq. (6) in step 3, explicit solutions of Eq. (3) can be obtained immediately depending on the Value $$\lambda .$$

### (2+1)-dimensional Heisenberg ferromagnetic spin chains equation

We found that a novel integrable NLS type equation in the dimension (2+1) governs the Heisenberg ferromagnetic spin chains equation

8 $$iu_{t} + a_{1} u_{xx} + a_{1} u_{yy} + a_{3} u_{xy} - a_{4} {\text{|u|}}^{{2}} {\text{u}} = {0}{\text{.}}$$

9 $${\text{Where}}\quad a_{1} = \gamma^{4} \left( {J + J_{2} } \right),\;a_{2} = \gamma^{4} \left( {J_{1} + J_{2} } \right),\;a_{3} = 2\gamma^{4} J_{2} ,\;a_{4} = 2\gamma^{4} A.$$

Here, the *γ* lattice parameter is, the adjacent interaction along the diagonal is ${J}_{2}$, the uniaxial crystal field anisotropy parameter is *A*, and *J* and ${J}_{1}$ are the coefficients of the bilinear exchange interactions along the *X*- and *Y*-directions, respectively. It is generally known that nonlinear evolution equations can be used to explain the nonlinear phenomena in fluid dynamics, nonlinear optics, Bose–Einstein condensates, biological molecules, chemical systems, etc. In the context of the NLSE, nonlinear wave propagation in optimism, and particularly fiber optics, has been extensively researched. The NLSE depicts how an envelope function changes over the course of an optical cycle, with gradual variations being the assumption.

Different traveling wave solutions to Equation are what we're interested in finding (1). In light of this, we select the following form for the complex envelope traveling wave solutions.

10 $$U\left( {x,y,t} \right) = f\left( \xi \right)e^{{i\varphi \left( {x,y,t} \right)}} ;$$

where

11 $$\xi = h_{1} x + h_{2} y - vt,$$

12 $$\varphi = k_{1} x + k_{2} y - wt.$$

In this case, the real amplitude function to be computed is A (ξ), ξ the traveling coordinate is, and the envelope’s phase is φ(x,y,t). Wave numbers in the x-direction and y-directions are represented by the parameters k_1_ and k_2_, group velocities of wave packets are represented by v, and pulse frequencies are represented by is. Equation (9) is substituted into Eq. (8), and the real and imaginary components are separated, resulting in

13 $$\left. \begin{gathered} \left( {w - a_{1} k_{1}^{2} - a_{2} k_{2}^{2} - a_{3} k_{1} k_{2} } \right)f + \left( {a_{1} h_{1}^{2} + a_{2} h_{2}^{2} + a_{3} h_{1} h_{2} } \right)f^{\prime\prime} - a_{4} f^{3} = 0 \hfill \\ \left( {2a_{1} k_{1} h_{1} + 2a_{2} k_{2} h_{2} + a_{3} \left( {k_{1} h_{2} + k_{2} h_{1} } \right) - v} \right)f^{\prime} = 0 \hfill \\ \end{gathered} \right\}.$$

In Set 1, Set 2, and Set 3, the constraint relations on the obtained results are as follows:

- Set 1: The constraint relations in Set 1 are given by: l_0_ = 0, l_1_ = √(2√(a_4_(a_1_h_1_^2^ + a_3_h_1_h_2_ + a_2_h_2_^2^)))/a_4_, and m_1_ = 0.
- Set 2: The constraint relations in Set 2 are given by: l_0_ = 0, m_1_ = √(2√(a_4_(a_1_h_1_^2^ + a_3_h_1_h_2_ + a_2_h_2_^2^)))/a_4_, and l_1_ = 0.
- Set 3: The constraint relations in Set 3 are given by: ${\text{l}}_{{0}} \, = \,0$, l_1_ = √(2√(a_4_(a_1_h_1_^2^ + a_3_h_1_h_2_ + a_2_h_2_^2^)))/a_4_, and m_1_ = √(2√(a_4_(a_1_h_1_^2^ + a_3_h_1_h_2_ + a_2_h_2_^2^)))/a_4_.

These constraint relations specify the values of l_0_, l_1_, and m_1_ in each set based on the given expressions. These constraints ensure that the trial solution satisfies the specified conditions and is a valid solution for the original equations.

Now consider a trial solution $f\left( \xi \right) = l_{0} + \sum\limits_{i = 1}^{N} {\left[ {l_{i} g\left( \xi \right)^{i} + m_{i} g\left( \xi \right)^{ - i} } \right]} .$ and using the above equation we get the following solution set,

### Solution set

**Set-1**

$$v = 2a_{1} k_{1} h_{1} + 2a_{2} k_{2} h_{2} + a_{3} k_{1} h_{2} + a_{3} k_{2} h_{1} \;w = a_{2} k_{2}^{2} - 2a_{1} h_{1}^{2} \lambda - 2a_{3} h_{1} h_{2} \lambda - 2a_{2} h_{2}^{2} \lambda + a_{1} k_{1}^{2} + a_{3} k_{1} h_{2}$$

$$l_{0} = 0,\;l_{1} = \pm \frac{{\sqrt 2 \sqrt {a_{4} \left( {a_{1} h_{1}^{2} + a_{3} h_{1} h_{2} + a_{2} h_{2}^{2} } \right)} }}{{a_{4} }},\;m{}_{1} = 0$$

*Case-1*: hyperbolic function (when $\lambda < 0$)

**A.**

$$g\left( \xi \right) = \frac{{\sqrt { - \left( {p^{2} + r^{2} } \right)\lambda } - p\sqrt { - \lambda } \cosh \left( {2\sqrt { - \lambda } \left( {\xi + q} \right)} \right)}}{{p\sinh \left( {2\sqrt { - \lambda } \left( {\xi + q} \right)} \right) + r}}$$

$$\begin{gathered} f\left( {x,y,t} \right) = l_{0} + l_{1} g\left( \xi \right) + m_{1} g\left( \xi \right)^{ - 1} \hfill \\ = \pm \frac{{\sqrt 2 \sqrt {a_{4} \left( {a_{1} h_{1}^{2} + a_{3} h_{1} h_{2} + a_{2} h_{2}^{2} } \right)} }}{{a_{4} }}.\frac{{\sqrt { - \left( {p^{2} + r^{2} } \right)\lambda } - p\sqrt { - \lambda } \cosh \left( {2\sqrt { - \lambda } \left( {\xi + q} \right)} \right)}}{{p\sinh \left( {2\sqrt { - \lambda } \left( {\xi + q} \right)} \right) + r}} \hfill \\ \end{gathered}$$

where $$\xi = h_{1} x + h_{2} y - vt = h_{1} x + h_{2} y - \left( {2a_{1} k_{1} h_{1} + 2a_{2} k_{2} h_{2} + a_{3} k_{1} h_{2} + a_{3} k_{2} h_{1} } \right)t$$.

The solution of Eq. (8) is

14 $$\begin{gathered} U_{1,1} \left( {x,y,t} \right) = fe^{ - i\varphi } = (l_{0} + l_{1} g\left( \xi \right) + m_{1} g\left( \xi \right)^{ - 1} )e^{ - i\varphi } \hfill \\ = \pm \frac{{\sqrt 2 \sqrt {a_{4} \left( {a_{1} h_{1}^{2} + a_{3} h_{1} h_{2} + a_{2} h_{2}^{2} } \right)} }}{{a_{4} }}.\frac{{\sqrt { - \left( {p^{2} + r^{2} } \right)\lambda } - p\sqrt { - \lambda } \cosh \left( {2\sqrt { - \lambda } \left( {\xi + q} \right)} \right)}}{{p\sinh \left( {2\sqrt { - \lambda } \left( {\xi + q} \right)} \right) + r}}.e^{ - i\varphi } \hfill \\ \end{gathered}$$

where $$\varphi = k_{1} x + k_{2} y - wt = k_{1} x + k_{2} y - \left( {a_{2} k_{2}^{2} - 2a_{1} h_{1}^{2} \lambda - 2a_{3} h_{1} h_{2} \lambda - 2a_{2} h_{2}^{2} \lambda + a_{1} k_{1}^{2} + a_{3} k_{1} h_{2} } \right)t$$.

**B.**

$$g\left( \xi \right) = \frac{{ - \sqrt { - \left( {p^{2} + r^{2} } \right)\lambda } - p\sqrt { - \lambda } \cosh \left( {2\sqrt { - \lambda } \left( {\xi + q} \right)} \right)}}{{p\sinh \left( {2\sqrt { - \lambda } \left( {\xi + q} \right)} \right) + r}}$$

$$\begin{gathered} f\left( {x,y,t} \right) = l_{0} + l_{1} g\left( \xi \right) + m_{1} g\left( \xi \right)^{ - 1} \hfill \\ = \pm \frac{{\sqrt 2 \sqrt {a_{4} \left( {a_{1} h_{1}^{2} + a_{3} h_{1} h_{2} + a_{2} h_{2}^{2} } \right)} }}{{a_{4} }}.\frac{{ - \sqrt { - \left( {p^{2} + r^{2} } \right)\lambda } - p\sqrt { - \lambda } \cosh \left( {2\sqrt { - \lambda } \left( {\xi + q} \right)} \right)}}{{p\sinh \left( {2\sqrt { - \lambda } \left( {\xi + q} \right)} \right) + r}}. \hfill \\ \end{gathered}$$

where $$\xi = h_{1} x + h_{2} y - vt = h_{1} x + h_{2} y - \left( {2a_{1} k_{1} h_{1} + 2a_{2} k_{2} h_{2} + a_{3} k_{1} h_{2} + a_{3} k_{2} h_{1} } \right)t$$.

The solution of Eq. (8) is

15 $$\begin{gathered} U_{1,2} = fe^{ - i\varphi } = (l_{0} + l_{1} g\left( \xi \right) + m_{1} g\left( \xi \right)^{ - 1} )e^{ - i\varphi } \hfill \\ = \pm \frac{{\sqrt 2 \sqrt {a_{4} \left( {a_{1} h_{1}^{2} + a_{3} h_{1} h_{2} + a_{2} h_{2}^{2} } \right)} }}{{a_{4} }}.\frac{{ - \sqrt { - \left( {p^{2} + r^{2} } \right)\lambda } - p\sqrt { - \lambda } \cosh \left( {2\sqrt { - \lambda } \left( {\xi + q} \right)} \right)}}{{p\sinh \left( {2\sqrt { - \lambda } \left( {\xi + q} \right)} \right) + r}}e^{ - i\varphi } \hfill \\ \end{gathered}$$

where $$\varphi = k_{1} x + k_{2} y - wt = k_{1} x + k_{2} y - \left( {a_{2} k_{2}^{2} - 2a_{1} h_{1}^{2} \lambda - 2a_{3} h_{1} h_{2} \lambda - 2a_{2} h_{2}^{2} \lambda + a_{1} k_{1}^{2} + a_{3} k_{1} h_{2} } \right)t$$.

**C.**

$$g\left( \xi \right) = \sqrt { - \lambda } + \frac{{2p\sqrt { - \lambda } }}{{p + \cosh \left( {2\sqrt { - \lambda } \left( {\xi + q} \right)} \right) - \sinh \left( {2\sqrt { - \lambda } \left( {\xi + q} \right)} \right)}}$$

$$\begin{gathered} f = l_{0} + l_{1} g\left( \xi \right) + m_{1} g\left( \xi \right)^{ - 1} \hfill \\ = \pm \frac{{\sqrt 2 \sqrt {a_{4} \left( {a_{1} h_{1}^{2} + a_{3} h_{1} h_{2} + a_{2} h_{2}^{2} } \right)} }}{{a_{4} }}\left( {\sqrt { - \lambda } + \frac{{2p\sqrt { - \lambda } }}{{p + \cosh \left( {2\sqrt { - \lambda } \left( {\xi + q} \right)} \right) - \sinh \left( {2\sqrt { - \lambda } \left( {\xi + q} \right)} \right)}}} \right) \hfill \\ \end{gathered}$$

where $$\xi = h_{1} x + h_{2} y - vt = h_{1} x + h_{2} y - \left( {2a_{1} k_{1} h_{1} + 2a_{2} k_{2} h_{2} + a_{3} k_{1} h_{2} + a_{3} k_{2} h_{1} } \right)t$$.

The solution of Eq. (8) is

16 $$\begin{gathered} U_{1,3} = fe^{ - i\varphi } = (l_{0} + l_{1} g\left( \xi \right) + m_{1} g\left( \xi \right)^{ - 1} )e^{ - i\varphi } \hfill \\ = \pm \frac{{\sqrt 2 \sqrt {a_{4} \left( {a_{1} h_{1}^{2} + a_{3} h_{1} h_{2} + a_{2} h_{2}^{2} } \right)} }}{{a_{4} }}\left( {\sqrt { - \lambda } + \frac{{2p\sqrt { - \lambda } }}{{p + \cosh \left( {2\sqrt { - \lambda } \left( {\xi + q} \right)} \right) - \sinh \left( {2\sqrt { - \lambda } \left( {\xi + q} \right)} \right)}}} \right)e^{ - i\varphi } \hfill \\ \end{gathered}$$

where $$\varphi = k_{1} x + k_{2} y - wt = k_{1} x + k_{2} y - \left( {a_{2} k_{2}^{2} - 2a_{1} h_{1}^{2} \lambda - 2a_{3} h_{1} h_{2} \lambda - 2a_{2} h_{2}^{2} \lambda + a_{1} k_{1}^{2} + a_{3} k_{1} h_{2} } \right)t$$.

**D.**

$$g\left( \xi \right) = - \sqrt { - \lambda } + \frac{{2p\sqrt { - \lambda } }}{{p + \cosh \left( {2\sqrt { - \lambda } \left( {\xi + q} \right)} \right) - \sinh \left( {2\sqrt { - \lambda } \left( {\xi + q} \right)} \right)}}$$

$$\begin{gathered} f = l_{0} + l_{1} g\left( \xi \right) + m_{1} g\left( \xi \right)^{ - 1} \hfill \\ = \pm \frac{{\sqrt 2 \sqrt {a_{4} \left( {a_{1} h_{1}^{2} + a_{3} h_{1} h_{2} + a_{2} h_{2}^{2} } \right)} }}{{a_{4} }}\left( { - \sqrt { - \lambda } + \frac{{2p\sqrt { - \lambda } }}{{p + \cosh \left( {2\sqrt { - \lambda } \left( {\xi + q} \right)} \right) - \sinh \left( {2\sqrt { - \lambda } \left( {\xi + q} \right)} \right)}}} \right) \hfill \\ \end{gathered}$$

where $$\xi = h_{1} x + h_{2} y - vt = h_{1} x + h_{2} y - \left( {2a_{1} k_{1} h_{1} + 2a_{2} k_{2} h_{2} + a_{3} k_{1} h_{2} + a_{3} k_{2} h_{1} } \right)t$$.

The solution of Eq. (8) is

17 $$\begin{gathered} U_{1,4} = fe^{ - i\varphi } = (l_{0} + l_{1} g\left( \xi \right) + m_{1} g\left( \xi \right)^{ - 1} )e^{ - i\varphi } \hfill \\ = \pm \frac{{\sqrt 2 \sqrt {a_{4} \left( {a_{1} h_{1}^{2} + a_{3} h_{1} h_{2} + a_{2} h_{2}^{2} } \right)} }}{{a_{4} }}\left( {\sqrt { - \lambda } + \frac{{2p\sqrt { - \lambda } }}{{p + \cosh \left( {2\sqrt { - \lambda } \left( {\xi + q} \right)} \right) - \sinh \left( {2\sqrt { - \lambda } \left( {\xi + q} \right)} \right)}}} \right)e^{ - i\varphi } \hfill \\ \end{gathered}$$

where $$\varphi = k_{1} x + k_{2} y - wt = k_{1} x + k_{2} y - \left( {a_{2} k_{2}^{2} - 2a_{1} h_{1}^{2} \lambda - 2a_{3} h_{1} h_{2} \lambda - 2a_{2} h_{2}^{2} \lambda + a_{1} k_{1}^{2} + a_{3} k_{1} h_{2} } \right)t$$.

*Case-02*: Trigonometric function (when $\lambda > 0$):

**A.**

$$g\left( \xi \right) = \frac{{\sqrt {\left( {p^{2} - r^{2} } \right)\lambda } - p\sqrt \lambda \cos \left( {2\sqrt \lambda \left( {\xi + q} \right)} \right)}}{{p\sin \left( {2\sqrt \lambda \left( {\xi + q} \right)} \right) + r}},$$

$$\begin{gathered} f = l_{0} + l_{1} g\left( \xi \right) + m_{1} g\left( \xi \right)^{ - 1} \hfill \\ = \pm \frac{{\sqrt 2 \sqrt {a_{4} \left( {a_{1} h_{1}^{2} + a_{3} h_{1} h_{2} + a_{2} h_{2}^{2} } \right)} }}{{a_{4} }}.\frac{{\sqrt {\left( {p^{2} - r^{2} } \right)\lambda } - p\sqrt \lambda \cos \left( {2\sqrt \lambda \left( {\xi + q} \right)} \right)}}{{p\sin \left( {2\sqrt \lambda \left( {\xi + q} \right)} \right) + r}} \hfill \\ \end{gathered}$$

where $$\xi = h_{1} x + h_{2} y - vt = h_{1} x + h_{2} y - \left( {2a_{1} k_{1} h_{1} + 2a_{2} k_{2} h_{2} + a_{3} k_{1} h_{2} + a_{3} k_{2} h_{1} } \right)t$$.

The solution of Eq. (8) is

18 $$\begin{gathered} U_{1,5} = fe^{ - i\varphi } = (l_{0} + l_{1} g\left( \xi \right) + m_{1} g\left( \xi \right)^{ - 1} )e^{ - i\varphi } \hfill \\ \left( { \pm \frac{{\sqrt 2 \sqrt {a_{4} \left( {a_{1} h_{1}^{2} + a_{3} h_{1} h_{2} + a_{2} h_{2}^{2} } \right)} }}{{a_{4} }}.\frac{{\sqrt {\left( {p^{2} - r^{2} } \right)\lambda } - p\sqrt \lambda \cos \left( {2\sqrt \lambda \left( {\xi + q} \right)} \right)}}{{p\sin \left( {2\sqrt \lambda \left( {\xi + q} \right)} \right) + r}}} \right)e^{ - i\varphi } \hfill \\ \end{gathered}$$

where $$\varphi = k_{1} x + k_{2} y - wt = k_{1} x + k_{2} y - \left( {a_{2} k_{2}^{2} - 2a_{1} h_{1}^{2} \lambda - 2a_{3} h_{1} h_{2} \lambda - 2a_{2} h_{2}^{2} \lambda + a_{1} k_{1}^{2} + a_{3} k_{1} h_{2} } \right)t$$.

**B.**

$$g\left( \xi \right) = \frac{{ - \sqrt {\left( {p^{2} - r^{2} } \right)\lambda } - p\sqrt \lambda \cos \left( {2\sqrt \lambda \left( {\xi + q} \right)} \right)}}{{p\sin \left( {2\sqrt \lambda \left( {\xi + q} \right)} \right) + r}},$$

$$\begin{gathered} f = l_{0} + l_{1} g\left( \xi \right) + m_{1} g\left( \xi \right)^{ - 1} \hfill \\ \pm \frac{{\sqrt 2 \sqrt {a_{4} \left( {a_{1} h_{1}^{2} + a_{3} h_{1} h_{2} + a_{2} h_{2}^{2} } \right)} }}{{a_{4} }}.\frac{{ - \sqrt {\left( {p^{2} - r^{2} } \right)\lambda } - p\sqrt \lambda \cos \left( {2\sqrt \lambda \left( {\xi + q} \right)} \right)}}{{p\sin \left( {2\sqrt \lambda \left( {\xi + q} \right)} \right) + r}} \hfill \\ \end{gathered}$$

where $$\xi = h_{1} x + h_{2} y - vt = h_{1} x + h_{2} y - \left( {2a_{1} k_{1} h_{1} + 2a_{2} k_{2} h_{2} + a_{3} k_{1} h_{2} + a_{3} k_{2} h_{1} } \right)t$$.

The solution of Eq. (8) is

19 $$\begin{gathered} U_{1,6} = fe^{ - i\varphi } = (l_{0} + l_{1} g\left( \xi \right) + m_{1} g\left( \xi \right)^{ - 1} )e^{ - i\varphi } \hfill \\ \left( { \pm \frac{{\sqrt 2 \sqrt {a_{4} \left( {a_{1} h_{1}^{2} + a_{3} h_{1} h_{2} + a_{2} h_{2}^{2} } \right)} }}{{a_{4} }}.\frac{{ - \sqrt {\left( {p^{2} - r^{2} } \right)\lambda } - p\sqrt \lambda \cos \left( {2\sqrt \lambda \left( {\xi + q} \right)} \right)}}{{p\sin \left( {2\sqrt \lambda \left( {\xi + q} \right)} \right) + r}}} \right)e^{ - i\iota } \hfill \\ \end{gathered}$$

where $$\varphi = k_{1} x + k_{2} y - wt = k_{1} x + k_{2} y - \left( {a_{2} k_{2}^{2} - 2a_{1} h_{1}^{2} \lambda - 2a_{3} h_{1} h_{2} \lambda - 2a_{2} h_{2}^{2} \lambda + a_{1} k_{1}^{2} + a_{3} k_{1} h_{2} } \right)t$$.

**C.**

$$g\left( \xi \right) = i\sqrt \lambda - \frac{2pi\sqrt \lambda }{{p + \cos \left( {2\sqrt \lambda \left( {\xi + q} \right)} \right) - i\sin \left( {2\sqrt \lambda \left( {\xi + q} \right)} \right)}}$$

$$\begin{gathered} f = l_{0} + l_{1} g\left( \xi \right) + m_{1} g\left( \xi \right)^{ - 1} \hfill \\ = \pm \frac{{\sqrt 2 \sqrt {a_{4} \left( {a_{1} h_{1}^{2} + a_{3} h_{1} h_{2} + a_{2} h_{2}^{2} } \right)} }}{{a_{4} }}\left( {i\sqrt \lambda - \frac{2pi\sqrt \lambda }{{p + \cos \left( {2\sqrt \lambda \left( {\xi + q} \right)} \right) - i\sin \left( {2\sqrt \lambda \left( {\xi + q} \right)} \right)}}} \right) \hfill \\ \end{gathered}$$

where $$\xi = h_{1} x + h_{2} y - vt = h_{1} x + h_{2} y - \left( {2a_{1} k_{1} h_{1} + 2a_{2} k_{2} h_{2} + a_{3} k_{1} h_{2} + a_{3} k_{2} h_{1} } \right)t$$.

The solution of Eq. (8) is

20 $$\begin{gathered} U_{1,7} = fe^{ - i\varphi } = (l_{0} + l_{1} g\left( \xi \right) + m_{1} g\left( \xi \right)^{ - 1} )e^{ - i\varphi } \hfill \\ = \left( { \pm \frac{{\sqrt 2 \sqrt {a_{4} \left( {a_{1} h_{1}^{2} + a_{3} h_{1} h_{2} + a_{2} h_{2}^{2} } \right)} }}{{a_{4} }}\left( {i\sqrt \lambda - \frac{2pi\sqrt \lambda }{{p + \cos \left( {2\sqrt \lambda \left( {\xi + q} \right)} \right) - i\sin \left( {2\sqrt \lambda \left( {\xi + q} \right)} \right)}}} \right)} \right)e^{ - i\varphi } \hfill \\ \end{gathered}$$

where $$\varphi = k_{1} x + k_{2} y - wt = k_{1} x + k_{2} y - \left( {a_{2} k_{2}^{2} - 2a_{1} h_{1}^{2} \lambda - 2a_{3} h_{1} h_{2} \lambda - 2a_{2} h_{2}^{2} \lambda + a_{1} k_{1}^{2} + a_{3} k_{1} h_{2} } \right)t$$.

**D.**

$$g\left( \xi \right) = - i\sqrt \lambda + \frac{2pi\sqrt \lambda }{{p + \cos \left( {2\sqrt \lambda \left( {\xi + q} \right)} \right) - i\sin \left( {2\sqrt \lambda \left( {\xi + q} \right)} \right)}}$$

$$\begin{gathered} f = l_{0} + l_{1} g\left( \xi \right) + m_{1} g\left( \xi \right)^{ - 1} \hfill \\ = \pm \frac{{\sqrt 2 \sqrt {a_{4} \left( {a_{1} h_{1}^{2} + a_{3} h_{1} h_{2} + a_{2} h_{2}^{2} } \right)} }}{{a_{4} }}\left( { - i\sqrt \lambda + \frac{2pi\sqrt \lambda }{{p + \cos \left( {2\sqrt \lambda \left( {\xi + q} \right)} \right) - i\sin \left( {2\sqrt \lambda \left( {\xi + q} \right)} \right)}}} \right) \hfill \\ \end{gathered}$$

where $$\xi = h_{1} x + h_{2} y - vt = h_{1} x + h_{2} y - \left( {2a_{1} k_{1} h_{1} + 2a_{2} k_{2} h_{2} + a_{3} k_{1} h_{2} + a_{3} k_{2} h_{1} } \right)t$$.

The solution of Eq. (8) is

21 $$\begin{gathered} U_{1,8} = fe^{ - i\varphi } = (l_{0} + l_{1} g\left( \xi \right) + m_{1} g\left( \xi \right)^{ - 1} )e^{ - i\varphi } \hfill \\ = \left( { \pm \frac{{\sqrt 2 \sqrt {a_{4} \left( {a_{1} h_{1}^{2} + a_{3} h_{1} h_{2} + a_{2} h_{2}^{2} } \right)} }}{{a_{4} }}\left( { - i\sqrt \lambda + \frac{2pi\sqrt \lambda }{{p + \cos \left( {2\sqrt \lambda \left( {\xi + q} \right)} \right) - i\sin \left( {2\sqrt \lambda \left( {\xi + q} \right)} \right)}}} \right)} \right)e^{ - i\varphi } \hfill \\ \end{gathered}$$

where $$\varphi = k_{1} x + k_{2} y - wt = k_{1} x + k_{2} y - \left( {a_{2} k_{2}^{2} - 2a_{1} h_{1}^{2} \lambda - 2a_{3} h_{1} h_{2} \lambda - 2a_{2} h_{2}^{2} \lambda + a_{1} k_{1}^{2} + a_{3} k_{1} h_{2} } \right)t$$.

*Case-03*: Rational function solutions (when $\lambda = 0$)

$$g\left( \xi \right) = \frac{1}{\xi + q}$$

$$\begin{gathered} f = l_{0} + l_{1} g\left( \xi \right) + m_{1} g\left( \xi \right)^{ - 1} \hfill \\ = \pm \frac{{\sqrt 2 \sqrt {a_{4} \left( {a_{1} h_{1}^{2} + a_{3} h_{1} h_{2} + a_{2} h_{2}^{2} } \right)} }}{{a_{4} }}.\frac{1}{\xi + q} \hfill \\ \end{gathered}$$

where $$\xi = h_{1} x + h_{2} y - vt = h_{1} x + h_{2} y - \left( {2a_{1} k_{1} h_{1} + 2a_{2} k_{2} h_{2} + a_{3} k_{1} h_{2} + a_{3} k_{2} h_{1} } \right)t$$.

The solution of Eq. (8) is

22 $$\begin{gathered} U_{1,9} = fe^{ - i\varphi } = (l_{0} + l_{1} g\left( \xi \right) + m_{1} g\left( \xi \right)^{ - 1} )e^{ - i\varphi } \hfill \\ = \left( { \pm \frac{{\sqrt 2 \sqrt {a_{4} \left( {a_{1} h_{1}^{2} + a_{3} h_{1} h_{2} + a_{2} h_{2}^{2} } \right)} }}{{a_{4} }}.\frac{1}{\xi + q}} \right)e^{ - i\varphi } \hfill \\ \end{gathered}$$

where $$\varphi = k_{1} x + k_{2} y - wt = k_{1} x + k_{2} y - \left( {a_{2} k_{2}^{2} - 2a_{1} h_{1}^{2} \lambda - 2a_{3} h_{1} h_{2} \lambda - 2a_{2} h_{2}^{2} \lambda + a_{1} k_{1}^{2} + a_{3} k_{1} h_{2} } \right)t$$.

**Set-2**

$$v = 2a_{1} k_{1} h_{1} + 2a_{2} k_{2} h_{2} + a_{3} k_{1} h_{2} + a_{3} k_{2} h_{1}$$

$$w = a_{2} k_{2}^{2} - 2a_{1} h_{1}^{2} \lambda - 2a_{3} h_{1} h_{2} \lambda - 2a_{2} h_{2}^{2} \lambda + a_{1} k_{1}^{2} + a_{3} k_{1} h_{2}$$

$$l_{0} = 0,\;m_{1} = \pm \frac{{\sqrt 2 \sqrt {a_{4} \left( {a_{1} h_{1}^{2} + a_{3} h_{1} h_{2} + a_{2} h_{2}^{2} } \right)} }}{{a_{4} }},\;l{}_{1} = 0$$

*Case-1*: hyperbolic function (when $\lambda < 0$)

**A.**

$$g\left( \xi \right) = \frac{{\sqrt { - \left( {p^{2} + r^{2} } \right)\lambda } - p\sqrt { - \lambda } \cosh \left( {2\sqrt { - \lambda } \left( {\xi + q} \right)} \right)}}{{p\sinh \left( {2\sqrt { - \lambda } \left( {\xi + q} \right)} \right) + r}}$$

$$\begin{gathered} f = l_{0} + l_{1} g\left( \xi \right) + m_{1} g\left( \xi \right)^{ - 1} \hfill \\ = m_{1} g\left( \xi \right)^{ - 1} = \pm \left( {\frac{{\sqrt 2 \sqrt {a_{4} \left( {a_{1} h_{1}^{2} + a_{3} h_{1} h_{2} + a_{2} h_{2}^{2} } \right)} }}{{a_{4} }}} \right).\left( {\frac{{p\sinh \left( {2\sqrt { - \lambda } \left( {\xi + q} \right)} \right) + r}}{{\sqrt { - \left( {p^{2} + r^{2} } \right)\lambda } - p\sqrt { - \lambda } \cosh \left( {2\sqrt { - \lambda } \left( {\xi + q} \right)} \right)}}} \right) \hfill \\ \end{gathered}$$

where $$\xi = h_{1} x + h_{2} y - vt = h_{1} x + h_{2} y - \left( {2a_{1} k_{1} h_{1} + 2a_{2} k_{2} h_{2} + a_{3} k_{1} h_{2} + a_{3} k_{2} h_{1} } \right)t$$.

The solution of Eq. (8) is

23 $$\begin{gathered} U_{2,1} = fe^{ - i\varphi } = (l_{0} + l_{1} g\left( \xi \right) + m_{1} g\left( \xi \right)^{ - 1} )e^{ - i\varphi } \hfill \\ = \pm \left( {\frac{{\sqrt 2 \sqrt {a_{4} \left( {a_{1} h_{1}^{2} + a_{3} h_{1} h_{2} + a_{2} h_{2}^{2} } \right)} }}{{a_{4} }}} \right).\left( {\frac{{p\sinh \left( {2\sqrt { - \lambda } \left( {\xi + q} \right)} \right) + r}}{{\sqrt { - \left( {p^{2} + r^{2} } \right)\lambda } - p\sqrt { - \lambda } \cosh \left( {2\sqrt { - \lambda } \left( {\xi + q} \right)} \right)}}} \right).e^{ - i\varphi } \hfill \\ \end{gathered}$$

where $$\varphi = k_{1} x + k_{2} y - wt = k_{1} x + k_{2} y - \left( {a_{2} k_{2}^{2} - 2a_{1} h_{1}^{2} \lambda - 2a_{3} h_{1} h_{2} \lambda - 2a_{2} h_{2}^{2} \lambda + a_{1} k_{1}^{2} + a_{3} k_{1} h_{2} } \right)t$$.

**B.**

$$g\left( \xi \right) = \frac{{ - \sqrt { - \left( {p^{2} + r^{2} } \right)\lambda } - p\sqrt { - \lambda } \cosh \left( {2\sqrt { - \lambda } \left( {\xi + q} \right)} \right)}}{{p\sinh \left( {2\sqrt { - \lambda } \left( {\xi + q} \right)} \right) + r}}$$

$$\begin{gathered} f = l_{0} + l_{1} g\left( \xi \right) + m_{1} g\left( \xi \right)^{ - 1} \hfill \\ = m_{1} g\left( \xi \right)^{ - 1} = \pm \left( {\frac{{\sqrt 2 \sqrt {a_{4} \left( {a_{1} h_{1}^{2} + a_{3} h_{1} h_{2} + a_{2} h_{2}^{2} } \right)} }}{{a_{4} }}} \right).\left( {\frac{{p\sinh \left( {2\sqrt { - \lambda } \left( {\xi + q} \right)} \right) + r}}{{ - \sqrt { - \left( {p^{2} + r^{2} } \right)\lambda } - p\sqrt { - \lambda } \cosh \left( {2\sqrt { - \lambda } \left( {\xi + q} \right)} \right)}}} \right) \hfill \\ \end{gathered}$$

where $$\xi = h_{1} x + h_{2} y - vt = h_{1} x + h_{2} y - \left( {2a_{1} k_{1} h_{1} + 2a_{2} k_{2} h_{2} + a_{3} k_{1} h_{2} + a_{3} k_{2} h_{1} } \right)t$$.

The solution of Eq. (8) is

24 $$\begin{gathered} U_{2,2} = fe^{ - i\varphi } = (l_{0} + l_{1} g\left( \xi \right) + m_{1} g\left( \xi \right)^{ - 1} )e^{ - i\varphi } \hfill \\ = \pm \left( {\frac{{\sqrt 2 \sqrt {a_{4} \left( {a_{1} h_{1}^{2} + a_{3} h_{1} h_{2} + a_{2} h_{2}^{2} } \right)} }}{{a_{4} }}} \right).\left( {\frac{{p\sinh \left( {2\sqrt { - \lambda } \left( {\xi + q} \right)} \right) + r}}{{\sqrt { - \left( {p^{2} + r^{2} } \right)\lambda } - p\sqrt { - \lambda } \cosh \left( {2\sqrt { - \lambda } \left( {\xi + q} \right)} \right)}}} \right).e^{ - i\varphi } \hfill \\ \end{gathered}$$

where $$\varphi = k_{1} x + k_{2} y - wt = k_{1} x + k_{2} y - \left( {a_{2} k_{2}^{2} - 2a_{1} h_{1}^{2} \lambda - 2a_{3} h_{1} h_{2} \lambda - 2a_{2} h_{2}^{2} \lambda + a_{1} k_{1}^{2} + a_{3} k_{1} h_{2} } \right)t$$.

**C.**

$$g\left( \xi \right) = \sqrt { - \lambda } + \frac{{2p\sqrt { - \lambda } }}{{p + \cosh \left( {2\sqrt { - \lambda } \left( {\xi + q} \right)} \right) - \sinh \left( {2\sqrt { - \lambda } \left( {\xi + q} \right)} \right)}}$$

$$\begin{gathered} f = l_{0} + l_{1} g\left( \xi \right) + m_{1} g\left( \xi \right)^{ - 1} \hfill \\ = \pm \left( {\frac{\sqrt 2 \sqrt \mu }{{a_{4} }}} \right).\left( {\frac{{p + \cosh \left( {2\sqrt { - \lambda } \left( {\xi + q} \right)} \right) - \sinh \left( {2\sqrt { - \lambda } \left( {\xi + q} \right)} \right)}}{{\left( {p + \cosh \left( {2\sqrt { - \lambda } \left( {\xi + q} \right)} \right) - \sinh \left( {2\sqrt { - \lambda } \left( {\xi + q} \right)} \right)} \right).\sqrt { - \lambda } + 2p\sqrt { - \lambda } }}} \right) \hfill \\ \end{gathered}$$

where $$\mu = a_{4} \left( {a_{1} h_{1}^{2} + a_{3} h_{1} h_{2} + a_{2} h_{2}^{2} } \right)$$.

And $$\xi = h_{1} x + h_{2} y - vt = h_{1} x + h_{2} y - \left( {2a_{1} k_{1} h_{1} + 2a_{2} k_{2} h_{2} + a_{3} k_{1} h_{2} + a_{3} k_{2} h_{1} } \right)t$$.

The solution of Eq. (8) is

25 $$\begin{gathered} U_{2,3} = fe^{ - i\varphi } = (l_{0} + l_{1} g\left( \xi \right) + m_{1} g\left( \xi \right)^{ - 1} )e^{ - i\varphi } \hfill \\ = \pm \left( {\frac{\sqrt 2 \sqrt \mu }{{a_{4} }}} \right).\left( {\frac{{p + \cosh \left( {2\sqrt { - \lambda } \left( {\xi + q} \right)} \right) - \sinh \left( {2\sqrt { - \lambda } \left( {\xi + q} \right)} \right)}}{{\left( {p + \cosh \left( {2\sqrt { - \lambda } \left( {\xi + q} \right)} \right) - \sinh \left( {2\sqrt { - \lambda } \left( {\xi + q} \right)} \right)} \right).\sqrt { - \lambda } + 2p\sqrt { - \lambda } }}} \right).e^{ - i\varphi } \hfill \\ \end{gathered}$$

where $$\mu = a_{4} \left( {a_{1} h_{1}^{2} + a_{3} h_{1} h_{2} + a_{2} h_{2}^{2} } \right)$$.

And $$\varphi = k_{1} x + k_{2} y - wt = k_{1} x + k_{2} y - \left( {a_{2} k_{2}^{2} - 2a_{1} h_{1}^{2} \lambda - 2a_{3} h_{1} h_{2} \lambda - 2a_{2} h_{2}^{2} \lambda + a_{1} k_{1}^{2} + a_{3} k_{1} h_{2} } \right)t$$.

**D.**

$$g\left( \xi \right) = - \sqrt { - \lambda } + \frac{{2p\sqrt { - \lambda } }}{{p + \cosh \left( {2\sqrt { - \lambda } \left( {\xi + q} \right)} \right) - \sinh \left( {2\sqrt { - \lambda } \left( {\xi + q} \right)} \right)}}$$

$$\begin{gathered} f = l_{0} + l_{1} g\left( \xi \right) + m_{1} g\left( \xi \right)^{ - 1} \hfill \\ = \pm \left( {\frac{\sqrt 2 \sqrt \mu }{{a_{4} }}} \right).\left( {\frac{{p + \cosh \left( {2\sqrt { - \lambda } \left( {\xi + q} \right)} \right) - \sinh \left( {2\sqrt { - \lambda } \left( {\xi + q} \right)} \right)}}{{ - \left( {p + \cosh \left( {2\sqrt { - \lambda } \left( {\xi + q} \right)} \right) - \sinh \left( {2\sqrt { - \lambda } \left( {\xi + q} \right)} \right)} \right).\sqrt { - \lambda } + 2p\sqrt { - \lambda } }}} \right) \hfill \\ \end{gathered}$$

where $$\mu = a_{4} \left( {a_{1} h_{1}^{2} + a_{3} h_{1} h_{2} + a_{2} h_{2}^{2} } \right)$$.

And $$\xi = h_{1} x + h_{2} y - vt = h_{1} x + h_{2} y - \left( {2a_{1} k_{1} h_{1} + 2a_{2} k_{2} h_{2} + a_{3} k_{1} h_{2} + a_{3} k_{2} h_{1} } \right)t$$.

The solution of Eq. (8) is

$$U_{2,4} = fe^{ - i\varphi } = (l_{0} + l_{1} g\left( \xi \right) + m_{1} g\left( \xi \right)^{ - 1} )e^{ - i\varphi }$$

26 $$= \pm \left( {\frac{\sqrt 2 \sqrt \mu }{{a_{4} }}} \right).\left( {\frac{{p + \cosh \left( {2\sqrt { - \lambda } \left( {\xi + q} \right)} \right) - \sinh \left( {2\sqrt { - \lambda } \left( {\xi + q} \right)} \right)}}{{ - \left( {p + \cosh \left( {2\sqrt { - \lambda } \left( {\xi + q} \right)} \right) - \sinh \left( {2\sqrt { - \lambda } \left( {\xi + q} \right)} \right)} \right).\sqrt { - \lambda } + 2p\sqrt { - \lambda } }}} \right).e^{ - i\varphi }$$

where $$\mu = a_{4} \left( {a_{1} h_{1}^{2} + a_{3} h_{1} h_{2} + a_{2} h_{2}^{2} } \right)$$.

And $$\varphi = k_{1} x + k_{2} y - wt = k_{1} x + k_{2} y - \left( {a_{2} k_{2}^{2} - 2a_{1} h_{1}^{2} \lambda - 2a_{3} h_{1} h_{2} \lambda - 2a_{2} h_{2}^{2} \lambda + a_{1} k_{1}^{2} + a_{3} k_{1} h_{2} } \right)t$$.

*Case-02*: Trigonometric function (when $\lambda > 0$):

**A.**

$$g\left( \xi \right) = \frac{{\sqrt {\left( {p^{2} - r^{2} } \right)\lambda } - p\sqrt \lambda \cos \left( {2\sqrt \lambda \left( {\xi + q} \right)} \right)}}{{p\sin \left( {2\sqrt \lambda \left( {\xi + q} \right)} \right) + r}},$$

$$\begin{gathered} f = l_{0} + l_{1} g\left( \xi \right) + m_{1} g\left( \xi \right)^{ - 1} \hfill \\ = \pm \left( {\frac{{\sqrt 2 \sqrt {a_{4} \left( {a_{1} h_{1}^{2} + a_{3} h_{1} h_{2} + a_{2} h_{2}^{2} } \right)} }}{{a_{4} }}} \right).\left( {\frac{{p\sin \left( {2\sqrt \lambda \left( {\xi + q} \right)} \right) + r}}{{\sqrt {\left( {p^{2} - r^{2} } \right)\lambda } - p\sqrt \lambda \cos \left( {2\sqrt \lambda \left( {\xi + q} \right)} \right)}}} \right) \hfill \\ \end{gathered}$$

where $$\xi = h_{1} x + h_{2} y - vt = h_{1} x + h_{2} y - \left( {2a_{1} k_{1} h_{1} + 2a_{2} k_{2} h_{2} + a_{3} k_{1} h_{2} + a_{3} k_{2} h_{1} } \right)t$$.

The solution of Eq. (8) is

27 $$\begin{gathered} U_{2,5} = fe^{ - i\varphi } = (l_{0} + l_{1} g\left( \xi \right) + m_{1} g\left( \xi \right)^{ - 1} )e^{ - i\varphi } \hfill \\ = \pm \left( {\frac{{\sqrt 2 \sqrt {a_{4} \left( {a_{1} h_{1}^{2} + a_{3} h_{1} h_{2} + a_{2} h_{2}^{2} } \right)} }}{{a_{4} }}} \right).\left( {\frac{{p\sin \left( {2\sqrt \lambda \left( {\xi + q} \right)} \right) + r}}{{\sqrt {\left( {p^{2} - r^{2} } \right)\lambda } - p\sqrt \lambda \cos \left( {2\sqrt \lambda \left( {\xi + q} \right)} \right)}}} \right)e^{ - i\varphi } \hfill \\ \end{gathered}$$

where $$\varphi = k_{1} x + k_{2} y - wt = k_{1} x + k_{2} y - \left( {a_{2} k_{2}^{2} - 2a_{1} h_{1}^{2} \lambda - 2a_{3} h_{1} h_{2} \lambda - 2a_{2} h_{2}^{2} \lambda + a_{1} k_{1}^{2} + a_{3} k_{1} h_{2} } \right)t$$.

**B.**

$$g\left( \xi \right) = \frac{{ - \sqrt {\left( {p^{2} - r^{2} } \right)\lambda } - p\sqrt \lambda \cos \left( {2\sqrt \lambda \left( {\xi + q} \right)} \right)}}{{p\sin \left( {2\sqrt \lambda \left( {\xi + q} \right)} \right) + r}},$$

$$\begin{gathered} f = l_{0} + l_{1} g\left( \xi \right) + m_{1} g\left( \xi \right)^{ - 1} \hfill \\ = \pm \left( {\frac{{\sqrt 2 \sqrt {a_{4} \left( {a_{1} h_{1}^{2} + a_{3} h_{1} h_{2} + a_{2} h_{2}^{2} } \right)} }}{{a_{4} }}} \right).\left( {\frac{{p\sin \left( {2\sqrt \lambda \left( {\xi + q} \right)} \right) + r}}{{ - \sqrt {\left( {p^{2} - r^{2} } \right)\lambda } - p\sqrt \lambda \cos \left( {2\sqrt \lambda \left( {\xi + q} \right)} \right)}}} \right) \hfill \\ \end{gathered}$$

where $$\xi = h_{1} x + h_{2} y - vt = h_{1} x + h_{2} y - \left( {2a_{1} k_{1} h_{1} + 2a_{2} k_{2} h_{2} + a_{3} k_{1} h_{2} + a_{3} k_{2} h_{1} } \right)t$$.

The solution of Eq. (8) is

28 $$\begin{gathered} U_{2,6} = fe^{ - i\varphi } = (l_{0} + l_{1} g\left( \xi \right) + m_{1} g\left( \xi \right)^{ - 1} )e^{ - i\varphi } \hfill \\ = \pm \left( {\frac{{\sqrt 2 \sqrt {a_{4} \left( {a_{1} h_{1}^{2} + a_{3} h_{1} h_{2} + a_{2} h_{2}^{2} } \right)} }}{{a_{4} }}} \right).\left( {\frac{{p\sin \left( {2\sqrt \lambda \left( {\xi + q} \right)} \right) + r}}{{ - \sqrt {\left( {p^{2} - r^{2} } \right)\lambda } - p\sqrt \lambda \cos \left( {2\sqrt \lambda \left( {\xi + q} \right)} \right)}}} \right).e^{ - i\varphi } \hfill \\ \end{gathered}$$

where $$\varphi = k_{1} x + k_{2} y - wt = k_{1} x + k_{2} y - \left( {a_{2} k_{2}^{2} - 2a_{1} h_{1}^{2} \lambda - 2a_{3} h_{1} h_{2} \lambda - 2a_{2} h_{2}^{2} \lambda + a_{1} k_{1}^{2} + a_{3} k_{1} h_{2} } \right)t$$.

**C.**

$$g\left( \xi \right) = i\sqrt \lambda - \frac{2pi\sqrt \lambda }{{p + \cos \left( {2\sqrt \lambda \left( {\xi + q} \right)} \right) - i\sin \left( {2\sqrt \lambda \left( {\xi + q} \right)} \right)}}$$

$$\begin{gathered} f = l_{0} + l_{1} g\left( \xi \right) + m_{1} g\left( \xi \right)^{ - 1} \hfill \\ = \pm \left( {\frac{{\sqrt 2 \sqrt {a_{4} \left( {a_{1} h_{1}^{2} + a_{3} h_{1} h_{2} + a_{2} h_{2}^{2} } \right)} }}{{a_{4} }}} \right).\left( {\frac{{p + \cosh \left( {2\sqrt \lambda \left( {\xi + q} \right)} \right) - i\sin \left( {2\sqrt \lambda \left( {\xi + q} \right)} \right)}}{{\left( {p + \cosh \left( {2\sqrt \lambda \left( {\xi + q} \right)} \right) - i\sin \left( {2\sqrt \lambda \left( {\xi + q} \right)} \right)} \right).i\sqrt \lambda - 2pi\sqrt \lambda }}} \right) \hfill \\ \end{gathered}$$

where $$\xi = h_{1} x + h_{2} y - vt = h_{1} x + h_{2} y - \left( {2a_{1} k_{1} h_{1} + 2a_{2} k_{2} h_{2} + a_{3} k_{1} h_{2} + a_{3} k_{2} h_{1} } \right)t$$.

The solution of Eq. (8) is

29 $$\begin{gathered} U_{2,7} = fe^{ - i\varphi } = (l_{0} + l_{1} g\left( \xi \right) + m_{1} g\left( \xi \right)^{ - 1} )e^{ - i\varphi } \hfill \\ = \pm \left( {\frac{{\sqrt 2 \sqrt {a_{4} \left( {a_{1} h_{1}^{2} + a_{3} h_{1} h_{2} + a_{2} h_{2}^{2} } \right)} }}{{a_{4} }}} \right).\left( {\frac{{p + \cosh \left( {2\sqrt \lambda \left( {\xi + q} \right)} \right) - i\sin \left( {2\sqrt \lambda \left( {\xi + q} \right)} \right)}}{{\left( {p + \cosh \left( {2\sqrt \lambda \left( {\xi + q} \right)} \right) - i\sin \left( {2\sqrt \lambda \left( {\xi + q} \right)} \right)} \right).i\sqrt \lambda - 2pi\sqrt \lambda }}} \right).e^{ - i\varphi } \hfill \\ \end{gathered}$$

where $$\varphi = k_{1} x + k_{2} y - wt = k_{1} x + k_{2} y - \left( {a_{2} k_{2}^{2} - 2a_{1} h_{1}^{2} \lambda - 2a_{3} h_{1} h_{2} \lambda - 2a_{2} h_{2}^{2} \lambda + a_{1} k_{1}^{2} + a_{3} k_{1} h_{2} } \right)t$$.

**D.**

$$g\left( \xi \right) = - i\sqrt \lambda + \frac{2pi\sqrt \lambda }{{p + \cos \left( {2\sqrt \lambda \left( {\xi + q} \right)} \right) - i\sin \left( {2\sqrt \lambda \left( {\xi + q} \right)} \right)}}$$

$$\begin{gathered} f = l_{0} + l_{1} g\left( \xi \right) + m_{1} g\left( \xi \right)^{ - 1} \hfill \\ = \pm \left( {\frac{{\sqrt 2 \sqrt {a_{4} \left( {a_{1} h_{1}^{2} + a_{3} h_{1} h_{2} + a_{2} h_{2}^{2} } \right)} }}{{a_{4} }}} \right).\left( {\frac{{p + \cosh \left( {2\sqrt \lambda \left( {\xi + q} \right)} \right) - i\sin \left( {2\sqrt \lambda \left( {\xi + q} \right)} \right)}}{{ - \left( {p + \cosh \left( {2\sqrt \lambda \left( {\xi + q} \right)} \right) - i\sin \left( {2\sqrt \lambda \left( {\xi + q} \right)} \right)} \right).i\sqrt \lambda + 2pi\sqrt \lambda }}} \right) \hfill \\ \end{gathered}$$

where $$\xi = h_{1} x + h_{2} y - vt = h_{1} x + h_{2} y - \left( {2a_{1} k_{1} h_{1} + 2a_{2} k_{2} h_{2} + a_{3} k_{1} h_{2} + a_{3} k_{2} h_{1} } \right)t$$.

The solution of Eq. (8) is

30 $$\begin{gathered} U_{2,8} = fe^{ - i\varphi } = (l_{0} + l_{1} g\left( \xi \right) + m_{1} g\left( \xi \right)^{ - 1} )e^{ - i\varphi } \hfill \\ = \pm \left( {\frac{\sqrt 2 \sqrt \mu }{{a_{4} }}} \right).\left( {\frac{{p + \cosh \left( {2\sqrt \lambda \left( {\xi + q} \right)} \right) - i\sin \left( {2\sqrt \lambda \left( {\xi + q} \right)} \right)}}{{ - \left( {p + \cosh \left( {2\sqrt \lambda \left( {\xi + q} \right)} \right) - i\sin \left( {2\sqrt \lambda \left( {\xi + q} \right)} \right)} \right).i\sqrt \lambda - 2pi\sqrt \lambda }}} \right).e^{ - i\varphi } \hfill \\ \end{gathered}$$

where $$\mu = a_{4} \left( {a_{1} h_{1}^{2} + a_{3} h_{1} h_{2} + a_{2} h_{2}^{2} } \right)$$.

And $$\varphi = k_{1} x + k_{2} y - wt = k_{1} x + k_{2} y - \left( {a_{2} k_{2}^{2} - 2a_{1} h_{1}^{2} \lambda - 2a_{3} h_{1} h_{2} \lambda - 2a_{2} h_{2}^{2} \lambda + a_{1} k_{1}^{2} + a_{3} k_{1} h_{2} } \right)t$$.

*Case-03*: Rational function solutions (when $\lambda = 0$)

$$g\left( \xi \right) = \frac{1}{\xi + q}$$

$$\begin{gathered} f = l_{0} + l_{1} g\left( \xi \right) + m_{1} g\left( \xi \right)^{ - 1} \hfill \\ = \pm \left( {\frac{{\sqrt 2 \sqrt {a_{4} \left( {a_{1} h_{1}^{2} + a_{3} h_{1} h_{2} + a_{2} h_{2}^{2} } \right)} }}{{a_{4} }}} \right).\left( {\xi + q} \right) \hfill \\ \end{gathered}$$

The solution of Eq. (8) is

31 $$\begin{gathered} U_{2,9} = fe^{ - i\varphi } = (l_{0} + l_{1} g\left( \xi \right) + m_{1} g\left( \xi \right)^{ - 1} )e^{ - i\varphi } \hfill \\ = \pm \left( {\frac{{\sqrt 2 \sqrt {a_{4} \left( {a_{1} h_{1}^{2} + a_{3} h_{1} h_{2} + a_{2} h_{2}^{2} } \right)} }}{{a_{4} }}} \right).\left( {\xi + q} \right).e^{ - i\varphi } \hfill \\ \end{gathered}$$

where $$\varphi = k_{1} x + k_{2} y - wt = k_{1} x + k_{2} y - \left( {a_{2} k_{2}^{2} - 2a_{1} h_{1}^{2} \lambda - 2a_{3} h_{1} h_{2} \lambda - 2a_{2} h_{2}^{2} \lambda + a_{1} k_{1}^{2} + a_{3} k_{1} h_{2} } \right)t$$.

**Set-3**

$$v = 2a_{1} k_{1} h_{1} + 2a_{2} k_{2} h_{2} + a_{3} k_{1} h_{2} + a_{3} k_{2} h_{1}$$

$$w = a_{2} k_{2}^{2} - 2a_{1} h_{1}^{2} \lambda - 2a_{3} h_{1} h_{2} \lambda - 2a_{2} h_{2}^{2} \lambda + a_{1} k_{1}^{2} + a_{3} k_{1} h_{2} + 3a_{4} \left( {a_{4} z^{2} - 2a_{1} h_{1}^{2} - 2a_{3} h_{1} h_{2} - 2a_{2} h_{2}^{2} } \right)\lambda$$

By solving,

$$w = \pm \frac{1}{{3\lambda a_{4} }}\left( {\sqrt 3 \left( {\lambda \left( \kappa \right)} \right)^{\frac{1}{2}} } \right)$$

where $$\kappa = 2a_{3} h_{1} h_{2} \lambda + 2a_{1} h_{1}^{2} \lambda - a_{2} k_{2}^{2} + 2a_{2} h_{2}^{2} \lambda - a_{1} k_{1}^{2} - a_{3} k_{1} h_{2} + 6a_{4} \lambda a_{1} h_{1}^{2} + 6a_{4} \lambda a_{3} h_{1} h_{2} + 6a_{4} \lambda a_{2} h_{2}^{2}$$.

Taking positive

$$w = \frac{1}{{3\lambda a_{4} }}\left( {\sqrt 3 \left( {\lambda \left( \kappa \right)} \right)^{\frac{1}{2}} } \right)$$

where $$\kappa = 2a_{3} h_{1} h_{2} \lambda + 2a_{1} h_{1}^{2} \lambda - a_{2} k_{2}^{2} + 2a_{2} h_{2}^{2} \lambda - a_{1} k_{1}^{2} - a_{3} k_{1} h_{2} + 6a_{4} \lambda a_{1} h_{1}^{2} + 6a_{4} \lambda a_{3} h_{1} h_{2} + 6a_{4} \lambda a_{2} h_{2}^{2}$$

$$m_{1} = \pm \frac{{\sqrt 2 \sqrt {a_{4} \left( {a_{1} h_{1}^{2} + a_{3} h_{1} h_{2} + a_{2} h_{2}^{2} } \right)} }}{{a_{4} }}$$

$$l_{1} = \pm \frac{{\sqrt 2 \sqrt {a_{4} \left( {a_{1} h_{1}^{2} + a_{3} h_{1} h_{2} + a_{2} h_{2}^{2} } \right)} }}{{a_{4} }}$$

$$l_{0} = 0$$

*Case-1*: hyperbolic function (when $\lambda < 0$)

**A.**

$$g\left( \xi \right) = \frac{{\sqrt { - \left( {p^{2} + r^{2} } \right)\lambda } - p\sqrt { - \lambda } \cosh \left( {2\sqrt { - \lambda } \left( {\xi + q} \right)} \right)}}{{p\sinh \left( {2\sqrt { - \lambda } \left( {\xi + q} \right)} \right) + r}}$$

$$\begin{gathered} f = l_{0} + l_{1} g\left( \xi \right) + m_{1} g\left( \xi \right)^{ - 1} \hfill \\ = \pm \frac{{\sqrt 2 \sqrt {a_{4} \left( {a_{1} h_{1}^{2} + a_{3} h_{1} h_{2} + a_{2} h_{2}^{2} } \right)} }}{{a_{4} }}.\left( \begin{gathered} \frac{{\sqrt { - \left( {p^{2} + r^{2} } \right)\lambda } - p\sqrt { - \lambda } \cosh \left( {2\sqrt { - \lambda } \left( {\xi + q} \right)} \right)}}{{p\sinh \left( {2\sqrt { - \lambda } \left( {\xi + q} \right)} \right) + r}} \hfill \\ + \frac{{p\sinh \left( {2\sqrt { - \lambda } \left( {\xi + q} \right)} \right) + r}}{{\sqrt { - \left( {p^{2} + r^{2} } \right)\lambda } - p\sqrt { - \lambda } \cosh \left( {2\sqrt { - \lambda } \left( {\xi + q} \right)} \right)}} \hfill \\ \end{gathered} \right) \hfill \\ \end{gathered}$$

where $$\xi = h_{1} x + h_{2} y - vt = h_{1} x + h_{2} y - \left( {2a_{1} k_{1} h_{1} + 2a_{2} k_{2} h_{2} + a_{3} k_{1} h_{2} + a_{3} k_{2} h_{1} } \right)t$$.

The solution of Eq. (8) is

32 $$\begin{gathered} U_{3,1} = fe^{ - i\varphi } = (l_{0} + l_{1} g\left( \xi \right) + m_{1} g\left( \xi \right)^{ - 1} )e^{ - i\varphi } \hfill \\ = \pm \frac{{\sqrt 2 \sqrt {a_{4} \left( {a_{1} h_{1}^{2} + a_{3} h_{1} h_{2} + a_{2} h_{2}^{2} } \right)} }}{{a_{4} }}.\left( \begin{gathered} \frac{{\sqrt { - \left( {p^{2} + r^{2} } \right)\lambda } - p\sqrt { - \lambda } \cosh \left( {2\sqrt { - \lambda } \left( {\xi + q} \right)} \right)}}{{p\sinh \left( {2\sqrt { - \lambda } \left( {\xi + q} \right)} \right) + r}} \hfill \\ + \frac{{p\sinh \left( {2\sqrt { - \lambda } \left( {\xi + q} \right)} \right) + r}}{{\sqrt { - \left( {p^{2} + r^{2} } \right)\lambda } - p\sqrt { - \lambda } \cosh \left( {2\sqrt { - \lambda } \left( {\xi + q} \right)} \right)}} \hfill \\ \end{gathered} \right).e^{ - i\varphi } \hfill \\ \end{gathered}$$

where $$\varphi = k_{1} x + k_{2} y - wt$$.

**B.**

$$g\left( \xi \right) = \frac{{ - \sqrt { - \left( {p^{2} + r^{2} } \right)\lambda } - p\sqrt { - \lambda } \cosh \left( {2\sqrt { - \lambda } \left( {\xi + q} \right)} \right)}}{{p\sinh \left( {2\sqrt { - \lambda } \left( {\xi + q} \right)} \right) + r}}$$

$$\begin{gathered} f = l_{0} + l_{1} g\left( \xi \right) + m_{1} g\left( \xi \right)^{ - 1} \hfill \\ = \pm \frac{{\sqrt 2 \sqrt {a_{4} \left( {a_{1} h_{1}^{2} + a_{3} h_{1} h_{2} + a_{2} h_{2}^{2} } \right)} }}{{a_{4} }}.\left( \begin{gathered} \frac{{ - \sqrt { - \left( {p^{2} + r^{2} } \right)\lambda } - p\sqrt { - \lambda } \cosh \left( {2\sqrt { - \lambda } \left( {\xi + q} \right)} \right)}}{{p\sinh \left( {2\sqrt { - \lambda } \left( {\xi + q} \right)} \right) + r}} \hfill \\ + \frac{{p\sinh \left( {2\sqrt { - \lambda } \left( {\xi + q} \right)} \right) + r}}{{ - \sqrt { - \left( {p^{2} + r^{2} } \right)\lambda } - p\sqrt { - \lambda } \cosh \left( {2\sqrt { - \lambda } \left( {\xi + q} \right)} \right)}} \hfill \\ \end{gathered} \right) \hfill \\ \end{gathered}$$

where $$\xi = h_{1} x + h_{2} y - vt = h_{1} x + h_{2} y - \left( {2a_{1} k_{1} h_{1} + 2a_{2} k_{2} h_{2} + a_{3} k_{1} h_{2} + a_{3} k_{2} h_{1} } \right)t$$.

The solution of Eq. (8) is

33 $$\begin{gathered} U_{3,2} = fe^{ - i\varphi } = (l_{0} + l_{1} g\left( \xi \right) + m_{1} g\left( \xi \right)^{ - 1} )e^{ - i\varphi } \hfill \\ = \pm \frac{{\sqrt 2 \sqrt {a_{4} \left( {a_{1} h_{1}^{2} + a_{3} h_{1} h_{2} + a_{2} h_{2}^{2} } \right)} }}{{a_{4} }}.\left( \begin{gathered} \frac{{ - \sqrt { - \left( {p^{2} + r^{2} } \right)\lambda } - p\sqrt { - \lambda } \cosh \left( {2\sqrt { - \lambda } \left( {\xi + q} \right)} \right)}}{{p\sinh \left( {2\sqrt { - \lambda } \left( {\xi + q} \right)} \right) + r}} \hfill \\ + \frac{{p\sinh \left( {2\sqrt { - \lambda } \left( {\xi + q} \right)} \right) + r}}{{ - \sqrt { - \left( {p^{2} + r^{2} } \right)\lambda } - p\sqrt { - \lambda } \cosh \left( {2\sqrt { - \lambda } \left( {\xi + q} \right)} \right)}} \hfill \\ \end{gathered} \right).e^{ - i\varphi } \hfill \\ \end{gathered}$$

where $$\varphi = k_{1} x + k_{2} y - wt$$.

**C.**

$$g\left( \xi \right) = \sqrt { - \lambda } + \frac{{2p\sqrt { - \lambda } }}{{p + \cosh \left( {2\sqrt { - \lambda } \left( {\xi + q} \right)} \right) - \sinh \left( {2\sqrt { - \lambda } \left( {\xi + q} \right)} \right)}}$$

$$\begin{gathered} f = l_{0} + l_{1} g\left( \xi \right) + m_{1} g\left( \xi \right)^{ - 1} \hfill \\ = \pm \frac{\sqrt 2 \sqrt \mu }{{a_{4} }}.\left( \begin{gathered} \sqrt { - \lambda } + \frac{{2p\sqrt { - \lambda } }}{{p + \cosh \left( {2\sqrt { - \lambda } \left( {\xi + q} \right)} \right) - \sinh \left( {2\sqrt { - \lambda } \left( {\xi + q} \right)} \right)}} \hfill \\ + \frac{{p + \cosh \left( {2\sqrt { - \lambda } \left( {\xi + q} \right)} \right) - \sinh \left( {2\sqrt { - \lambda } \left( {\xi + q} \right)} \right)}}{{\left( {p + \cosh \left( {2\sqrt { - \lambda } \left( {\xi + q} \right)} \right) - \sinh \left( {2\sqrt { - \lambda } \left( {\xi + q} \right)} \right)} \right).\sqrt { - \lambda } + 2p\sqrt { - \lambda } }} \hfill \\ \end{gathered} \right). \hfill \\ \end{gathered}$$

where $$\mu = a_{4} \left( {a_{1} h_{1}^{2} + a_{3} h_{1} h_{2} + a_{2} h_{2}^{2} } \right)$$.

And $$\xi = h_{1} x + h_{2} y - vt = h_{1} x + h_{2} y - \left( {2a_{1} k_{1} h_{1} + 2a_{2} k_{2} h_{2} + a_{3} k_{1} h_{2} + a_{3} k_{2} h_{1} } \right)t$$.

The solution of Eq. (8) is

34 $$\begin{gathered} U_{3,3} = fe^{ - i\varphi } = (l_{0} + l_{1} g\left( \xi \right) + m_{1} g\left( \xi \right)^{ - 1} )e^{ - i\varphi } \hfill \\ = \pm \frac{\sqrt 2 \sqrt \mu }{{a_{4} }}.\left( \begin{gathered} \sqrt { - \lambda } + \frac{{2p\sqrt { - \lambda } }}{{p + \cosh \left( {2\sqrt { - \lambda } \left( {\xi + q} \right)} \right) - \sinh \left( {2\sqrt { - \lambda } \left( {\xi + q} \right)} \right)}} \hfill \\ + \frac{{p + \cosh \left( {2\sqrt { - \lambda } \left( {\xi + q} \right)} \right) - \sinh \left( {2\sqrt { - \lambda } \left( {\xi + q} \right)} \right)}}{{\left( {p + \cosh \left( {2\sqrt { - \lambda } \left( {\xi + q} \right)} \right) - \sinh \left( {2\sqrt { - \lambda } \left( {\xi + q} \right)} \right)} \right).\sqrt { - \lambda } + 2p\sqrt { - \lambda } }} \hfill \\ \end{gathered} \right).e^{ - i\varphi } \hfill \\ \end{gathered}$$

where $$\mu = a_{4} \left( {a_{1} h_{1}^{2} + a_{3} h_{1} h_{2} + a_{2} h_{2}^{2} } \right)$$.

And $$\varphi = k_{1} x + k_{2} y - wt$$.

**D.**

$$g\left( \xi \right) = - \sqrt { - \lambda } + \frac{{2p\sqrt { - \lambda } }}{{p + \cosh \left( {2\sqrt { - \lambda } \left( {\xi + q} \right)} \right) - \sinh \left( {2\sqrt { - \lambda } \left( {\xi + q} \right)} \right)}}$$

$$\begin{gathered} f = l_{0} + l_{1} g\left( \xi \right) + m_{1} g\left( \xi \right)^{ - 1} \hfill \\ = \pm \frac{\sqrt 2 \sqrt \mu }{{a_{4} }}.\left( \begin{gathered} - \sqrt { - \lambda } + \frac{{2p\sqrt { - \lambda } }}{{p + \cosh \left( {2\sqrt { - \lambda } \left( {\xi + q} \right)} \right) - \sinh \left( {2\sqrt { - \lambda } \left( {\xi + q} \right)} \right)}} \hfill \\ + \frac{{p + \cosh \left( {2\sqrt { - \lambda } \left( {\xi + q} \right)} \right) - \sinh \left( {2\sqrt { - \lambda } \left( {\xi + q} \right)} \right)}}{{ - \left( {p + \cosh \left( {2\sqrt { - \lambda } \left( {\xi + q} \right)} \right) - \sinh \left( {2\sqrt { - \lambda } \left( {\xi + q} \right)} \right)} \right).\sqrt { - \lambda } + 2p\sqrt { - \lambda } }} \hfill \\ \end{gathered} \right) \hfill \\ \end{gathered}$$

where $$\mu = a_{4} \left( {a_{1} h_{1}^{2} + a_{3} h_{1} h_{2} + a_{2} h_{2}^{2} } \right)$$.

And $$\xi = h_{1} x + h_{2} y - vt = h_{1} x + h_{2} y - \left( {2a_{1} k_{1} h_{1} + 2a_{2} k_{2} h_{2} + a_{3} k_{1} h_{2} + a_{3} k_{2} h_{1} } \right)t$$.

The solution of Eq. (8) is

35 $$\begin{gathered} U_{4,4} = fe^{ - i\varphi } = (l_{0} + l_{1} g\left( \xi \right) + m_{1} g\left( \xi \right)^{ - 1} )e^{ - i\varphi } \hfill \\ = \pm \frac{\sqrt 2 \sqrt \mu }{{a_{4} }}.\left( \begin{gathered} - \sqrt { - \lambda } + \frac{{2p\sqrt { - \lambda } }}{{p + \cosh \left( {2\sqrt { - \lambda } \left( {\xi + q} \right)} \right) - \sinh \left( {2\sqrt { - \lambda } \left( {\xi + q} \right)} \right)}} \hfill \\ + \frac{{p + \cosh \left( {2\sqrt { - \lambda } \left( {\xi + q} \right)} \right) - \sinh \left( {2\sqrt { - \lambda } \left( {\xi + q} \right)} \right)}}{{ - \left( {p + \cosh \left( {2\sqrt { - \lambda } \left( {\xi + q} \right)} \right) - \sinh \left( {2\sqrt { - \lambda } \left( {\xi + q} \right)} \right)} \right).\sqrt { - \lambda } + 2p\sqrt { - \lambda } }} \hfill \\ \end{gathered} \right).e^{ - i\varphi } \hfill \\ \end{gathered}$$

where $$\mu = a_{4} \left( {a_{1} h_{1}^{2} + a_{3} h_{1} h_{2} + a_{2} h_{2}^{2} } \right)$$ and $$\varphi = k_{1} x + k_{2} y - wt$$.

*Case-02*: Trigonometric function (when $\lambda > 0$):

**A.**

$$g\left( \xi \right) = \frac{{\sqrt {\left( {p^{2} - r^{2} } \right)\lambda } - p\sqrt \lambda \cos \left( {2\sqrt \lambda \left( {\xi + q} \right)} \right)}}{{p\sin \left( {2\sqrt \lambda \left( {\xi + q} \right)} \right) + r}},$$

$$\begin{gathered} f = l_{0} + l_{1} g\left( \xi \right) + m_{1} g\left( \xi \right)^{ - 1} \hfill \\ = \pm \frac{{\sqrt 2 \sqrt {a_{4} \left( {a_{1} h_{1}^{2} + a_{3} h_{1} h_{2} + a_{2} h_{2}^{2} } \right)} }}{{a_{4} }}.\left( \begin{gathered} \frac{{\sqrt {\left( {p^{2} - r^{2} } \right)\lambda } - p\sqrt \lambda \cos \left( {2\sqrt \lambda \left( {\xi + q} \right)} \right)}}{{p\sin \left( {2\sqrt \lambda \left( {\xi + q} \right)} \right) + r}} \hfill \\ + \frac{{p\sin \left( {2\sqrt \lambda \left( {\xi + q} \right)} \right) + r}}{{\sqrt {\left( {p^{2} - r^{2} } \right)\lambda } - p\sqrt \lambda \cos \left( {2\sqrt \lambda \left( {\xi + q} \right)} \right)}} \hfill \\ \end{gathered} \right) \hfill \\ \end{gathered}$$

where $$\xi = h_{1} x + h_{2} y - vt = h_{1} x + h_{2} y - \left( {2a_{1} k_{1} h_{1} + 2a_{2} k_{2} h_{2} + a_{3} k_{1} h_{2} + a_{3} k_{2} h_{1} } \right)t$$.

The solution of Eq. (8) is

36 $$\begin{gathered} U_{3,5} = fe^{ - i\varphi } = (l_{0} + l_{1} g\left( \xi \right) + m_{1} g\left( \xi \right)^{ - 1} )e^{ - i\varphi } \hfill \\ = \pm \frac{{\sqrt 2 \sqrt {a_{4} \left( {a_{1} h_{1}^{2} + a_{3} h_{1} h_{2} + a_{2} h_{2}^{2} } \right)} }}{{a_{4} }}.\left( \begin{gathered} \frac{{\sqrt {\left( {p^{2} - r^{2} } \right)\lambda } - p\sqrt \lambda \cos \left( {2\sqrt \lambda \left( {\xi + q} \right)} \right)}}{{p\sin \left( {2\sqrt \lambda \left( {\xi + q} \right)} \right) + r}} \hfill \\ + \frac{{p\sin \left( {2\sqrt \lambda \left( {\xi + q} \right)} \right) + r}}{{\sqrt {\left( {p^{2} - r^{2} } \right)\lambda } - p\sqrt \lambda \cos \left( {2\sqrt \lambda \left( {\xi + q} \right)} \right)}} \hfill \\ \end{gathered} \right).e^{ - i\varphi } \hfill \\ \end{gathered}$$

where $$\varphi = k_{1} x + k_{2} y - wt$$.

**B.**

$$g\left( \xi \right) = \frac{{ - \sqrt {\left( {p^{2} - r^{2} } \right)\lambda } - p\sqrt \lambda \cos \left( {2\sqrt \lambda \left( {\xi + q} \right)} \right)}}{{p\sin \left( {2\sqrt \lambda \left( {\xi + q} \right)} \right) + r}},$$

$$\begin{gathered} f = l_{0} + l_{1} g\left( \xi \right) + m_{1} g\left( \xi \right)^{ - 1} \hfill \\ = \pm \frac{{\sqrt 2 \sqrt {a_{4} \left( {a_{1} h_{1}^{2} + a_{3} h_{1} h_{2} + a_{2} h_{2}^{2} } \right)} }}{{a_{4} }}.\left( \begin{gathered} \frac{{ - \sqrt {\left( {p^{2} - r^{2} } \right)\lambda } - p\sqrt \lambda \cos \left( {2\sqrt \lambda \left( {\xi + q} \right)} \right)}}{{p\sin \left( {2\sqrt \lambda \left( {\xi + q} \right)} \right) + r}} \hfill \\ + \frac{{p\sin \left( {2\sqrt \lambda \left( {\xi + q} \right)} \right) + r}}{{ - \sqrt {\left( {p^{2} - r^{2} } \right)\lambda } - p\sqrt \lambda \cos \left( {2\sqrt \lambda \left( {\xi + q} \right)} \right)}} \hfill \\ \end{gathered} \right) \hfill \\ \end{gathered}$$

where $$\xi = h_{1} x + h_{2} y - vt = h_{1} x + h_{2} y - \left( {2a_{1} k_{1} h_{1} + 2a_{2} k_{2} h_{2} + a_{3} k_{1} h_{2} + a_{3} k_{2} h_{1} } \right)t$$.

The solution of Eq. (8) is

37 $$\begin{gathered} U_{3,6} = fe^{ - i\varphi } = (l_{0} + l_{1} g\left( \xi \right) + m_{1} g\left( \xi \right)^{ - 1} )e^{ - i\varphi } \hfill \\ = \pm \frac{{\sqrt 2 \sqrt {a_{4} \left( {a_{1} h_{1}^{2} + a_{3} h_{1} h_{2} + a_{2} h_{2}^{2} } \right)} }}{{a_{4} }}.\left( \begin{gathered} \frac{{ - \sqrt {\left( {p^{2} - r^{2} } \right)\lambda } - p\sqrt \lambda \cos \left( {2\sqrt \lambda \left( {\xi + q} \right)} \right)}}{{p\sin \left( {2\sqrt \lambda \left( {\xi + q} \right)} \right) + r}} \hfill \\ + \frac{{p\sin \left( {2\sqrt \lambda \left( {\xi + q} \right)} \right) + r}}{{ - \sqrt {\left( {p^{2} - r^{2} } \right)\lambda } - p\sqrt \lambda \cos \left( {2\sqrt \lambda \left( {\xi + q} \right)} \right)}} \hfill \\ \end{gathered} \right).e^{ - i\varphi } \hfill \\ \end{gathered}$$

where $$\varphi = k_{1} x + k_{2} y - wt = k_{1} x + k_{2} y - \left( {a_{2} k_{2}^{2} - 2a_{1} h_{1}^{2} \lambda - 2a_{3} h_{1} h_{2} \lambda - 2a_{2} h_{2}^{2} \lambda + a_{1} k_{1}^{2} + a_{3} k_{1} h_{2} } \right)t$$.

**C.**

$$g\left( \xi \right) = i\sqrt \lambda - \frac{2pi\sqrt \lambda }{{p + \cos \left( {2\sqrt \lambda \left( {\xi + q} \right)} \right) - i\sin \left( {2\sqrt \lambda \left( {\xi + q} \right)} \right)}}$$

$$\begin{gathered} f = l_{0} + l_{1} g\left( \xi \right) + m_{1} g\left( \xi \right)^{ - 1} \hfill \\ = \pm \frac{\sqrt 2 \sqrt \mu }{{a_{4} }}\left( \begin{gathered} i\sqrt \lambda - \frac{2pi\sqrt \lambda }{{p + \cosh \left( {2\sqrt \lambda \left( {\xi + q} \right)} \right) - i\sin \left( {2\sqrt \lambda \left( {\xi + q} \right)} \right)}} \hfill \\ + \frac{{p + \cosh \left( {2\sqrt \lambda \left( {\xi + q} \right)} \right) - i\sin \left( {2\sqrt \lambda \left( {\xi + q} \right)} \right)}}{{\left( {p + \cosh \left( {2\sqrt \lambda \left( {\xi + q} \right)} \right) - i\sin \left( {2\sqrt \lambda \left( {\xi + q} \right)} \right)} \right).i\sqrt \lambda - 2pi\sqrt \lambda }} \hfill \\ \end{gathered} \right) \hfill \\ \end{gathered}$$

where $$\mu = a_{4} \left( {a_{1} h_{1}^{2} + a_{3} h_{1} h_{2} + a_{2} h_{2}^{2} } \right)$$.

And $$\xi = h_{1} x + h_{2} y - vt = h_{1} x + h_{2} y - \left( {2a_{1} k_{1} h_{1} + 2a_{2} k_{2} h_{2} + a_{3} k_{1} h_{2} + a_{3} k_{2} h_{1} } \right)t$$.

The solution of Eq. (8) is

38 $$\begin{gathered} U_{3,7} = fe^{ - i\varphi } = (l_{0} + l_{1} g\left( \xi \right) + m_{1} g\left( \xi \right)^{ - 1} )e^{ - i\varphi } \hfill \\ = \pm \frac{\sqrt 2 \sqrt \mu }{{a_{4} }}\left( \begin{gathered} i\sqrt \lambda - \frac{2pi\sqrt \lambda }{{p + \cosh \left( {2\sqrt \lambda \left( {\xi + q} \right)} \right) - i\sin \left( {2\sqrt \lambda \left( {\xi + q} \right)} \right)}} \hfill \\ + \frac{{p + \cosh \left( {2\sqrt \lambda \left( {\xi + q} \right)} \right) - i\sin \left( {2\sqrt \lambda \left( {\xi + q} \right)} \right)}}{{\left( {p + \cosh \left( {2\sqrt \lambda \left( {\xi + q} \right)} \right) - i\sin \left( {2\sqrt \lambda \left( {\xi + q} \right)} \right)} \right).i\sqrt \lambda - 2pi\sqrt \lambda }} \hfill \\ \end{gathered} \right)e^{ - i\varphi } \hfill \\ \end{gathered}$$

where $$\mu = a_{4} \left( {a_{1} h_{1}^{2} + a_{3} h_{1} h_{2} + a_{2} h_{2}^{2} } \right)$$.

And $$\varphi = k_{1} x + k_{2} y - wt$$.

**D.**

$$g\left( \xi \right) = - i\sqrt \lambda + \frac{2pi\sqrt \lambda }{{p + \cos \left( {2\sqrt \lambda \left( {\xi + q} \right)} \right) - i\sin \left( {2\sqrt \lambda \left( {\xi + q} \right)} \right)}}$$

$$\begin{gathered} f = l_{0} + l_{1} g\left( \xi \right) + m_{1} g\left( \xi \right)^{ - 1} \hfill \\ = \pm \frac{\sqrt 2 \sqrt \mu }{{a_{4} }}\left( \begin{gathered} - i\sqrt \lambda + \frac{2pi\sqrt \lambda }{{p + \cosh \left( {2\sqrt \lambda \left( {\xi + q} \right)} \right) - i\sin \left( {2\sqrt \lambda \left( {\xi + q} \right)} \right)}} \hfill \\ + \frac{{p + \cosh \left( {2\sqrt \lambda \left( {\xi + q} \right)} \right) - i\sin \left( {2\sqrt \lambda \left( {\xi + q} \right)} \right)}}{{ - \left( {p + \cosh \left( {2\sqrt \lambda \left( {\xi + q} \right)} \right) - i\sin \left( {2\sqrt \lambda \left( {\xi + q} \right)} \right)} \right).i\sqrt \lambda + 2pi\sqrt \lambda }} \hfill \\ \end{gathered} \right) \hfill \\ \end{gathered}$$

where $$\mu = a_{4} \left( {a_{1} h_{1}^{2} + a_{3} h_{1} h_{2} + a_{2} h_{2}^{2} } \right)$$.

And $$\xi = h_{1} x + h_{2} y - vt = h_{1} x + h_{2} y - \left( {2a_{1} k_{1} h_{1} + 2a_{2} k_{2} h_{2} + a_{3} k_{1} h_{2} + a_{3} k_{2} h_{1} } \right)t$$.

The solution of Eq. (8) is

39 $$\begin{gathered} U_{3,8} = fe^{ - i\varphi } = (l_{0} + l_{1} g\left( \xi \right) + m_{1} g\left( \xi \right)^{ - 1} )e^{ - i\varphi } \hfill \\ = \pm \frac{\sqrt 2 \sqrt \mu }{{a_{4} }}\left( \begin{gathered} - i\sqrt \lambda + \frac{ - 2pi\sqrt \lambda }{{p + \cosh \left( {2\sqrt \lambda \left( {\xi + q} \right)} \right) - i\sin \left( {2\sqrt \lambda \left( {\xi + q} \right)} \right)}} \hfill \\ + \frac{{p + \cosh \left( {2\sqrt \lambda \left( {\xi + q} \right)} \right) - i\sin \left( {2\sqrt \lambda \left( {\xi + q} \right)} \right)}}{{ - \left( {p + \cosh \left( {2\sqrt \lambda \left( {\xi + q} \right)} \right) - i\sin \left( {2\sqrt \lambda \left( {\xi + q} \right)} \right)} \right).i\sqrt \lambda - 2pi\sqrt \lambda }} \hfill \\ \end{gathered} \right).e^{ - i\varphi } \hfill \\ \end{gathered}$$

where $$\mu = a_{4} \left( {a_{1} h_{1}^{2} + a_{3} h_{1} h_{2} + a_{2} h_{2}^{2} } \right)$$.

And $$\varphi = k_{1} x + k_{2} y - wt$$.

*Case-03*: Rational function solutions (when $\lambda = 0$)

$$g\left( \xi \right) = \frac{1}{\xi + q}$$

$$\begin{gathered} f = l_{0} + l_{1} g\left( \xi \right) + m_{1} g\left( \xi \right)^{ - 1} \hfill \\ = \pm \frac{{\sqrt 2 \sqrt {a_{4} \left( {a_{1} h_{1}^{2} + a_{3} h_{1} h_{2} + a_{2} h_{2}^{2} } \right)} }}{{a_{4} }}.\left( {\frac{1}{\xi + q} + \xi + q} \right) \hfill \\ \end{gathered}$$

where $$\xi = h_{1} x + h_{2} y - vt = h_{1} x + h_{2} y - \left( {2a_{1} k_{1} h_{1} + 2a_{2} k_{2} h_{2} + a_{3} k_{1} h_{2} + a_{3} k_{2} h_{1} } \right)t$$.

The solution of Eq. (8) is

40 $$\begin{gathered} U_{3,9} = fe^{ - i\varphi } = (l_{0} + l_{1} g\left( \xi \right) + m_{1} g\left( \xi \right)^{ - 1} )e^{ - i\varphi } \hfill \\ = \pm \frac{{\sqrt 2 \sqrt {a_{4} \left( {a_{1} h_{1}^{2} + a_{3} h_{1} h_{2} + a_{2} h_{2}^{2} } \right)} }}{{a_{4} }}.\left( {\frac{1}{\xi + q} + \xi + q} \right).e^{ = i\varphi } \hfill \\ \end{gathered}$$

where $$\varphi = k_{1} x + k_{2} y - wt$$.

## Graphical representation of (2+1) dimensional Heisenberg ferromagnetic spin chains equation

In this section, we plotted the figure by using mathematical Maple software, some three dimensional, two dimensional and some contour plots of the above equation are presented below: From the results of perturbation analysis, we found that the periodic (see Figs. 1, 2, 3, 4, 5, 6, 7, 8, 9, 10, 11, 12, 13, 14 and 15) solution of the stability of soliton. We also investigate 2-D, 3-D shapes for our analytical equation which are shown separately. These results may have potential applications in magnetic memory and recording devices.

**Figure 1:**
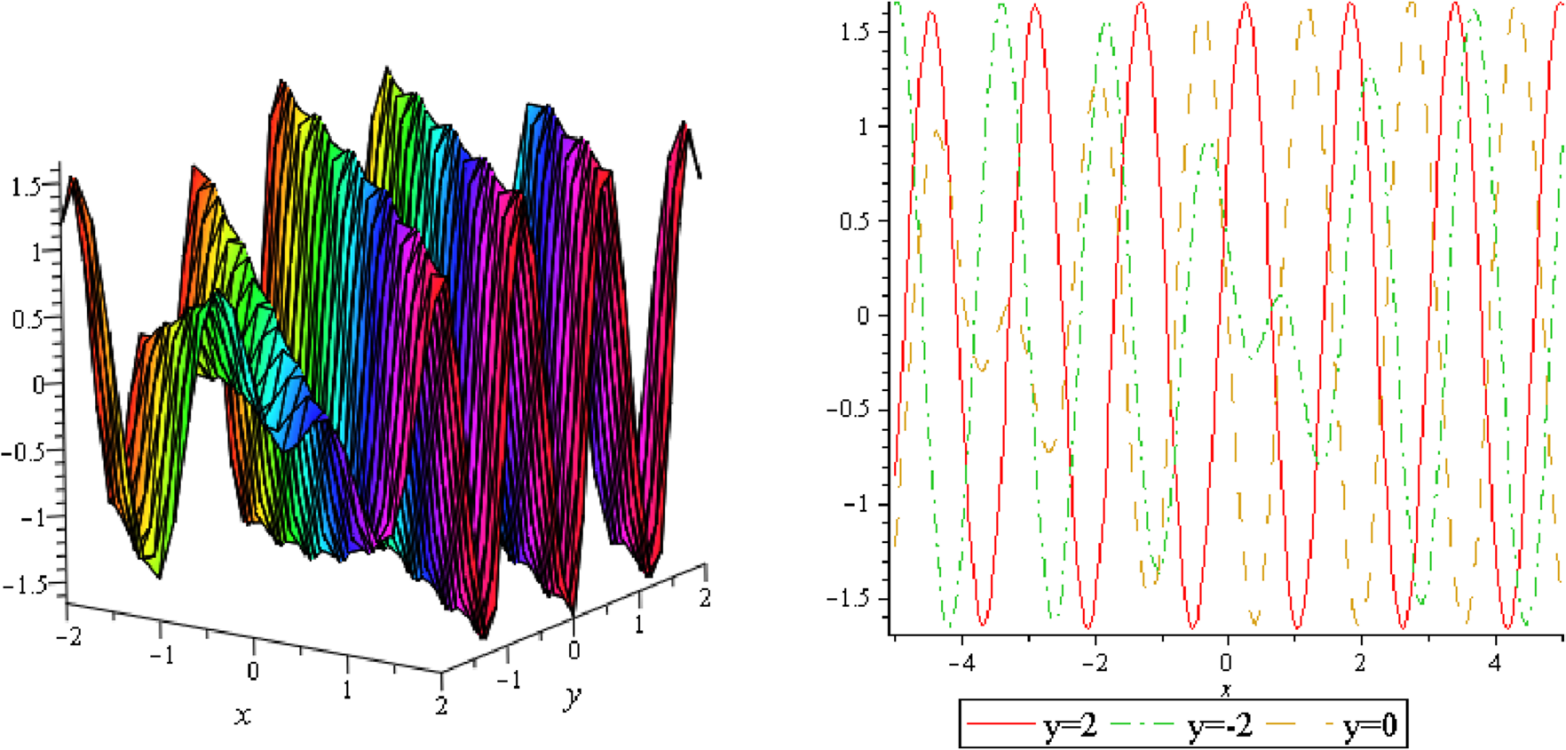
Periodic shape of $U_{1,1}$ for $a_{1} = 1$, $a_{2} = 2$, $a_{3} = 1$, $a_{4} = 4$, $k_{1} = 4$, $k_{5} = 5$, $h_{1} = \frac{1}{2}$, $h_{2} = 1$, $p = 1$, $q = 2$, $r = 1$, $\lambda = - 2$, $t = 0$ within the interval $- 2 \le x \le 2$ and $- 2 \le y \le 2$.

**Figure 2:**
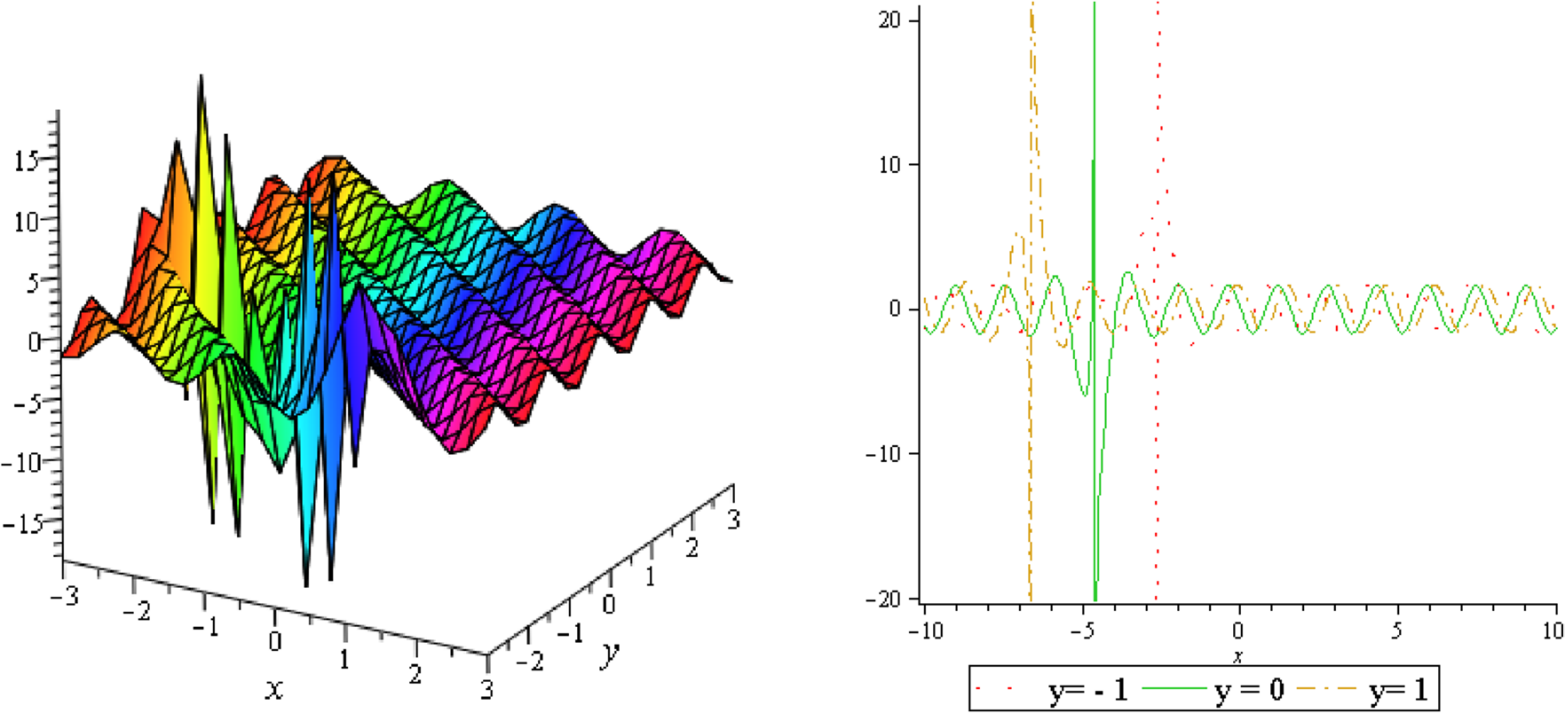
Periodic shape of $U_{1,2}$ for $a_{1} = 1$, $a_{2} = 2$, $a_{3} = 1$, $a_{4} = 4$, $k_{1} = 4$, $k_{5} = 5$, $h_{1} = \frac{1}{2}$, $h_{2} = 1$, $p = 1$, $q = 2$, $r = 1$, $\lambda = - 2$, $t = 0$ within the interval $- 3 \le x \le 3$ and $- 3 \le y \le 3$.

**Figure 3:**
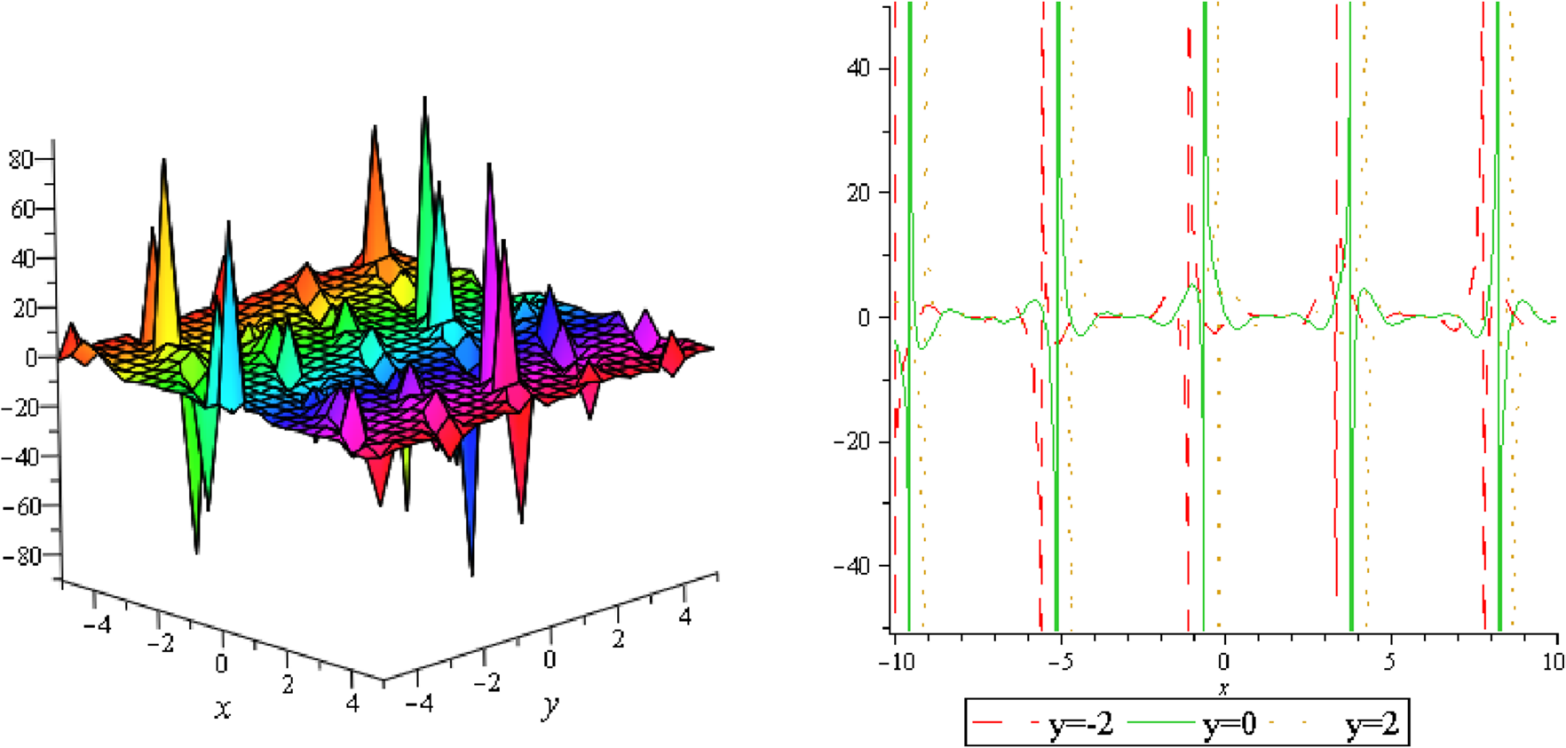
Periodic shape of $U_{1,5}$ for $a_{1} = 1$, $a_{2} = 2$, $a_{3} = 1$, $a_{4} = 4$, $k_{1} = 4$, $k_{5} = 5$, $h_{1} = \frac{1}{2}$, $h_{2} = 1$, $p = 1$, $q = 2$, $r = 1$, $\lambda = 2$, $t = 0$ within the interval $- 5 \le x \le 5$ and $- 5 \le y \le 5$.

**Figure 4:**
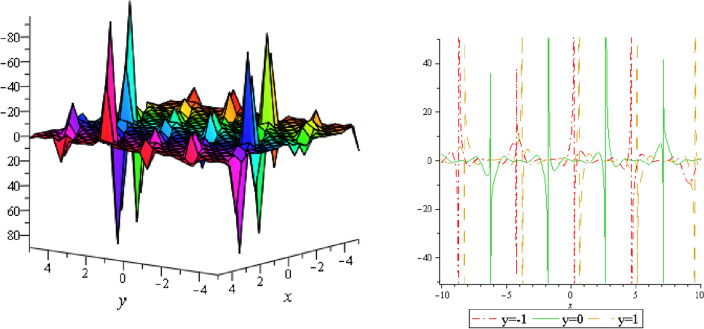
Periodic shape of $U_{1,7}$ for $a_{1} = 1$, $a_{2} = 2$, $a_{3} = 1$, $a_{4} = 4$, $k_{1} = 4$, $k_{5} = 5$, $h_{1} = \frac{1}{2}$, $h_{2} = 1$, $p = 1$, $q = 2$, $r = 1$, $\lambda = 2$, $t = 0$ within the interval $- 5 \le x \le 5$ and $- 5 \le y \le 5$.

**Figure 5:**
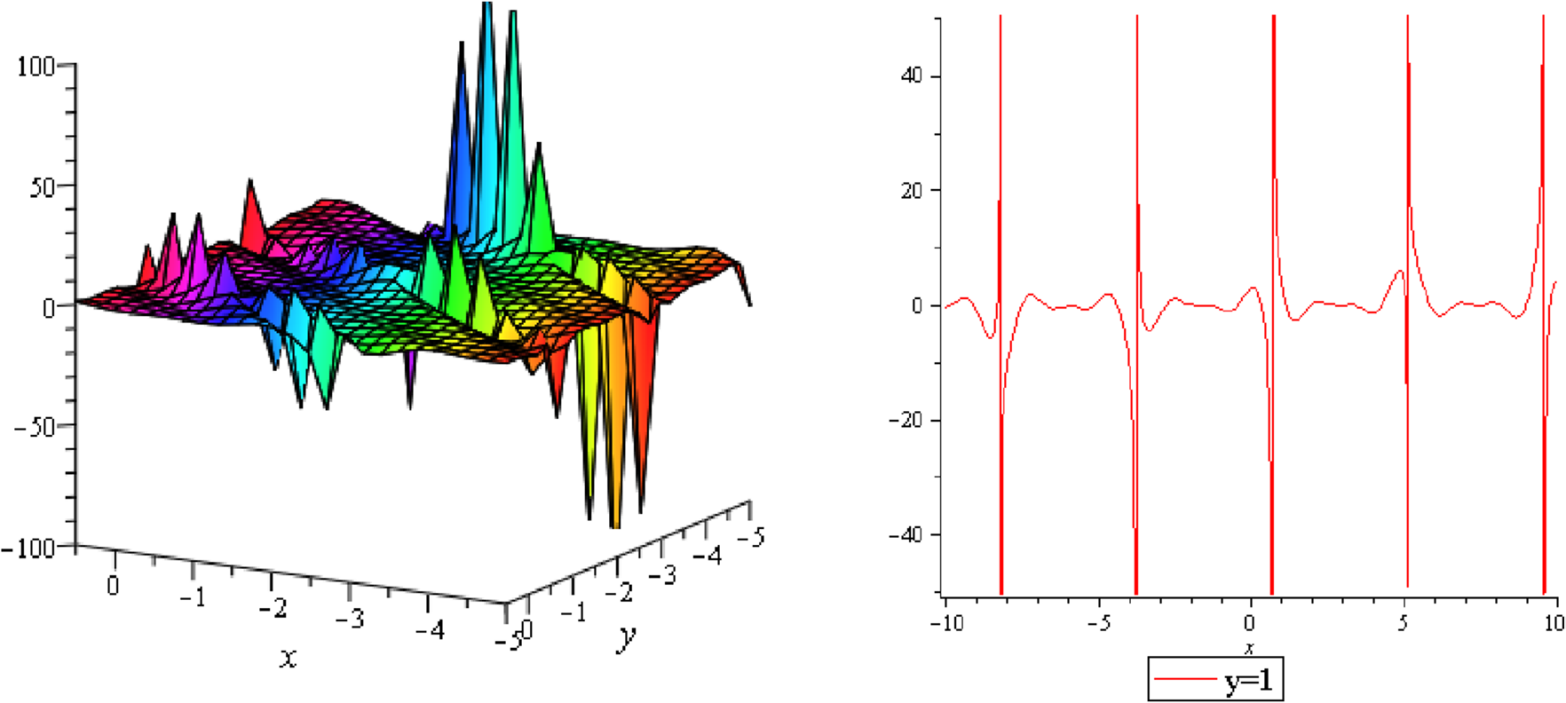
Periodic shape of $U_{1,8}$ for $a_{1} = 1$, $a_{2} = 2$, $a_{3} = 1$, $a_{4} = 4$, $k_{1} = 4$, $k_{5} = 5$, $h_{1} = \frac{1}{2}$, $h_{2} = 1$, $p = 1$, $q = 2$, $r = 1$, $\lambda = 2$, $t = 0$ within the interval $- 5 \le x \le 5$ and $- 5 \le y \le 5$.

**Figure 6:**
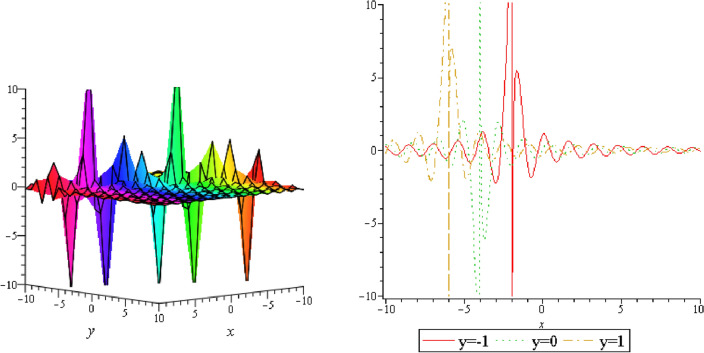
Periodic shape of $U_{1,7}$ for $a_{1} = 1$, $a_{2} = 2$, $a_{3} = 1$, $a_{4} = 4$, $k_{1} = 4$, $k_{5} = 5$, $h_{1} = \frac{1}{2}$, $h_{2} = 1$, $p = 1$, $q = 2$, $r = 1$, $\lambda = 0$, $t = 0$ within the interval $- 10 \le x \le 10$ and $- 10 \le y \le 10$.

**Figure 7:**
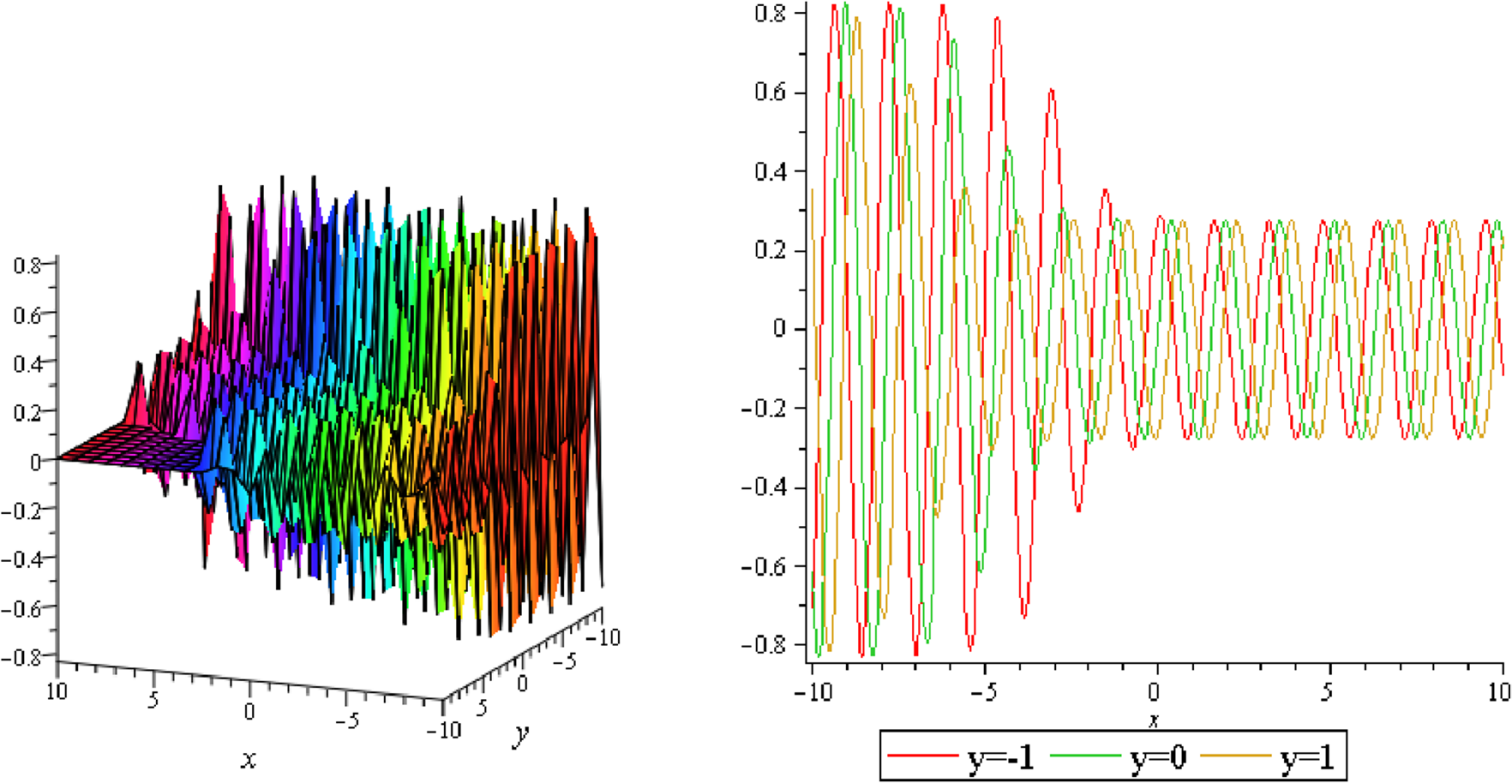
Periodic shape of $U_{2,3}$ for $a_{1} = 1$, $a_{2} = 2$, $a_{3} = 1$, $a_{4} = 4$, $k_{1} = 4$, $k_{5} = 5$, $h_{1} = \frac{1}{2}$, $h_{2} = 1$, $p = 1$, $q = 2$, $r = 1$, $\lambda = - 2$, $t = 0$ within the interval $- 10 \le x \le 10$ and $- 10 \le y \le 10$.

**Figure 8:**
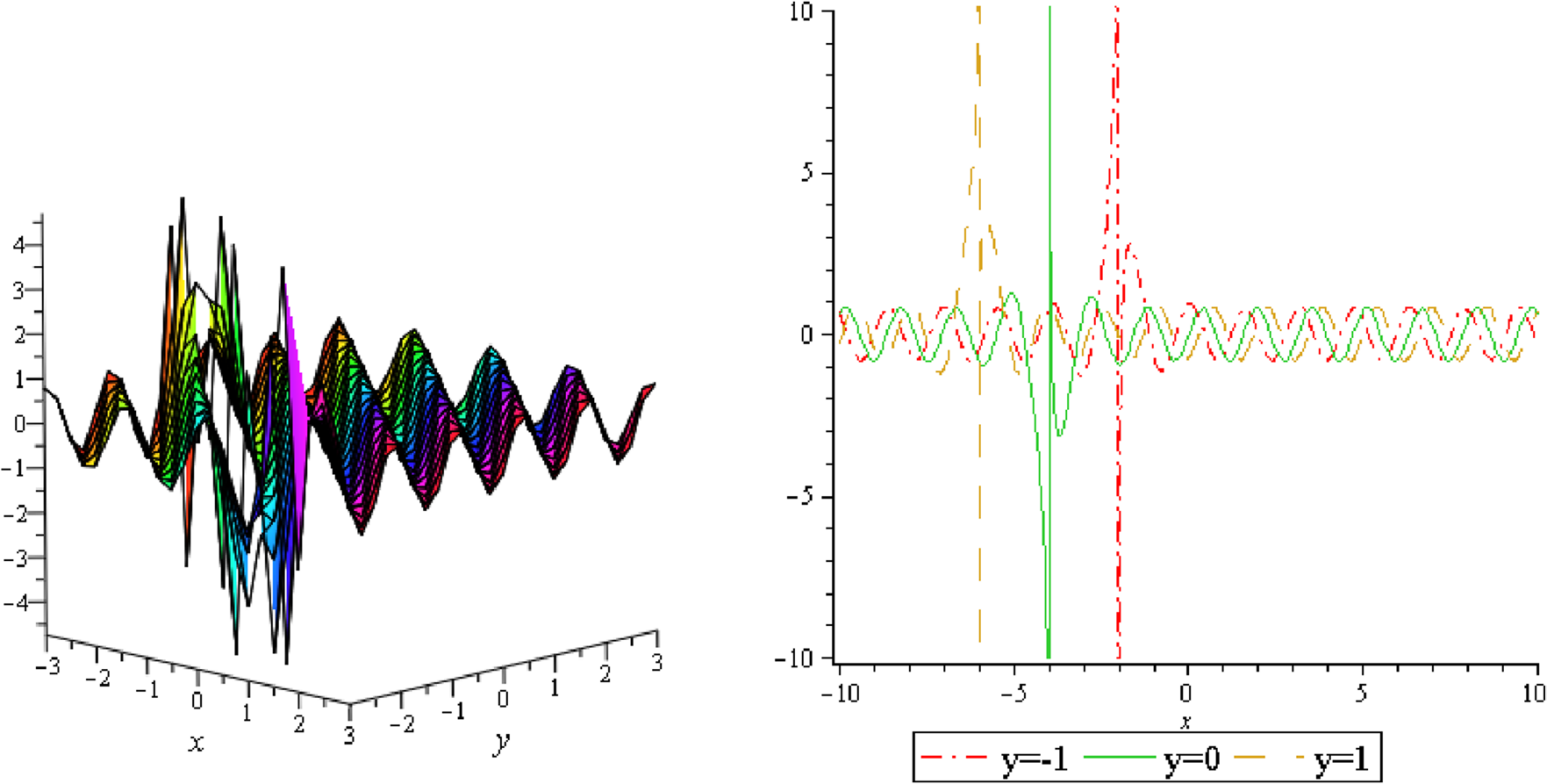
Periodic shape of $U_{2,3}$ for $a_{1} = 1$, $a_{2} = 2$, $a_{3} = 1$, $a_{4} = 4$, $k_{1} = 4$, $k_{5} = 5$, $h_{1} = \frac{1}{2}$, $h_{2} = 1$, $p = 1$, $q = 2$, $r = 1$, $\lambda = - 2$, $t = 0$ within the interval $- 3 \le x \le 3$ and $- 3 \le y \le 3$.

**Figure 9:**
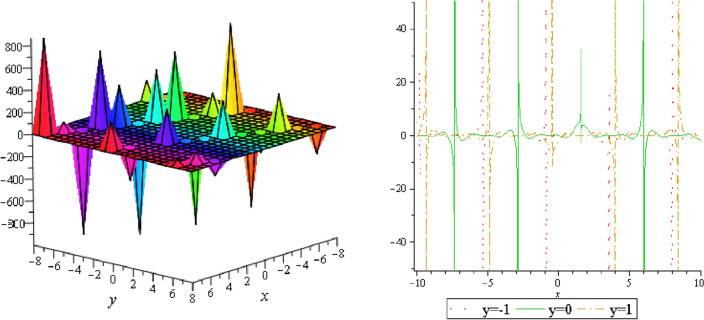
Periodic shape of $U_{2,3}$ for $a_{1} = 1$, $a_{2} = 2$, $a_{3} = 1$, $a_{4} = 4$, $k_{1} = 4$, $k_{5} = 5$, $h_{1} = \frac{1}{2}$, $h_{2} = 1$, $p = 1$, $q = 2$, $r = 1$, $\lambda = 2$, $t = 0$ within the interval $- 8 \le x \le 8$ and $- 8 \le y \le 8$.

**Figure 10:**
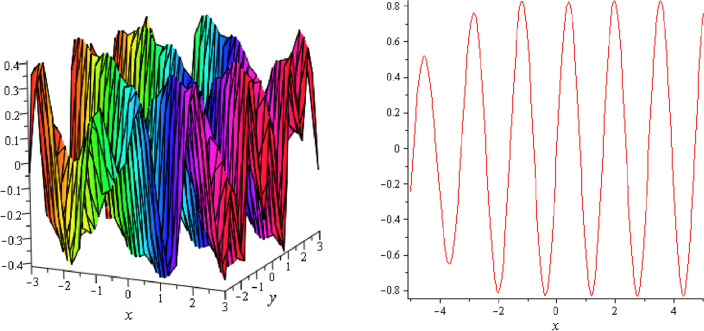
Periodic shape of $U_{2,7}$ for $a_{1} = 1$, $a_{2} = 2$, $a_{3} = 1$, $a_{4} = 4$, $k_{1} = 4$, $k_{5} = 5$, $h_{1} = \frac{1}{2}$, $h_{2} = 1$, $p = 1$, $q = 2$, $r = 1$, $\lambda = 2$, $t = 0$ within the interval $- 8 \le x \le 8$ and $- 8 \le y \le 8$.

**Figure 11:**
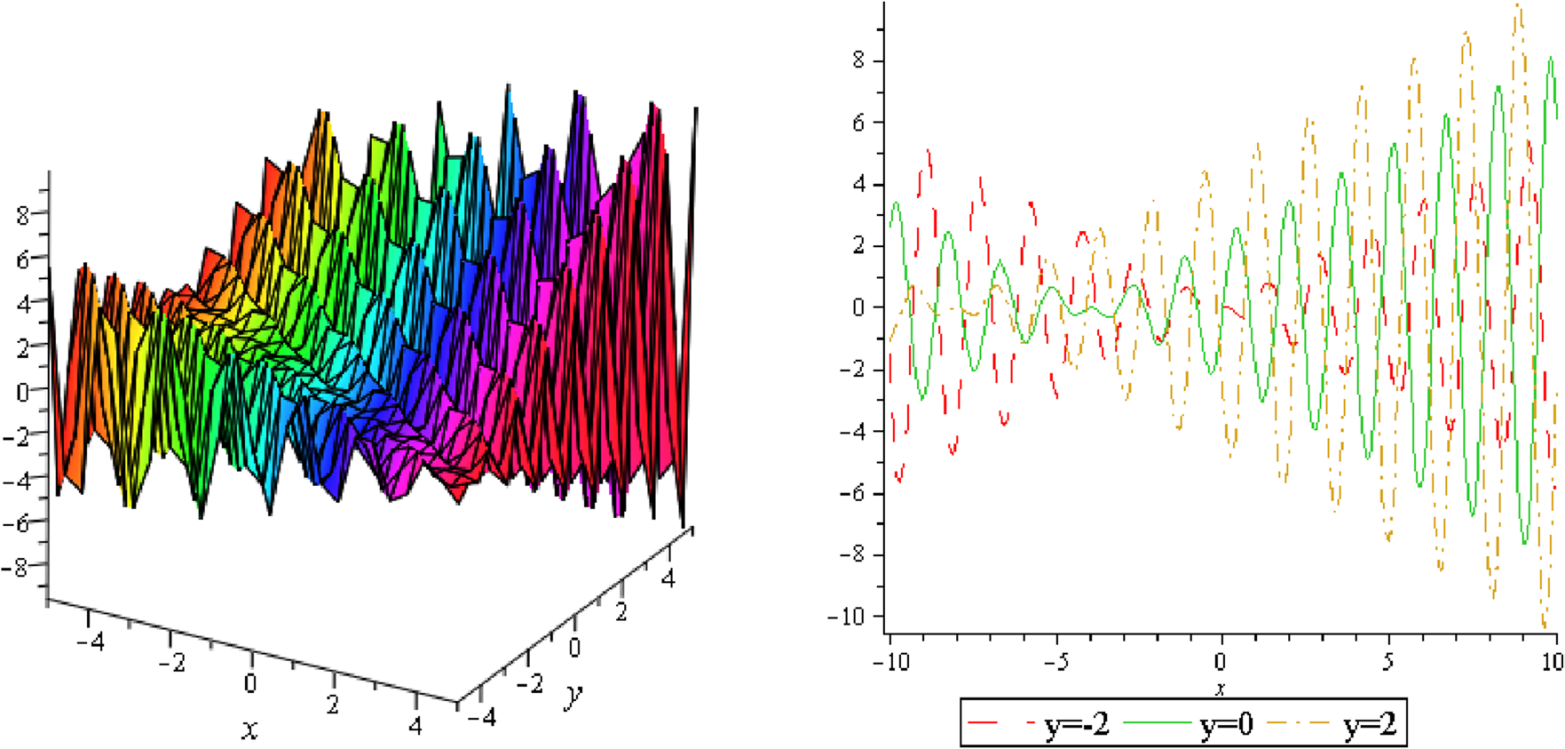
Periodic shape of $U_{2,9}$ for $a_{1} = 1$, $a_{2} = 2$, $a_{3} = 1$, $a_{4} = 4$, $k_{1} = 4$, $k_{5} = 5$, $h_{1} = \frac{1}{2}$, $h_{2} = 1$, $p = 1$, $q = 2$, $r = 1$, $\lambda = 0$, $t = 0$ within the interval $- 5 \le x \le 5$ and $- 5 \le y \le 5$.

**Figure 12:**
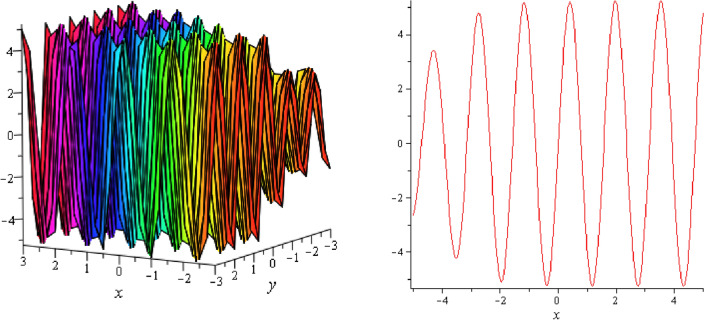
Periodic shape of $U_{3,3}$ for $a_{1} = 1$, $a_{2} = 2$, $a_{3} = 1$, $a_{4} = 4$, $k_{1} = 4$, $k_{5} = 5$, $h_{1} = \frac{1}{2}$, $h_{2} = 1$, $p = 1$, $q = 2$, $r = 1$, $\lambda = - 2$, $t = 0$ within the interval $- 3 \le x \le 3$ and $- 3 \le y \le 3$.

**Figure 13:**
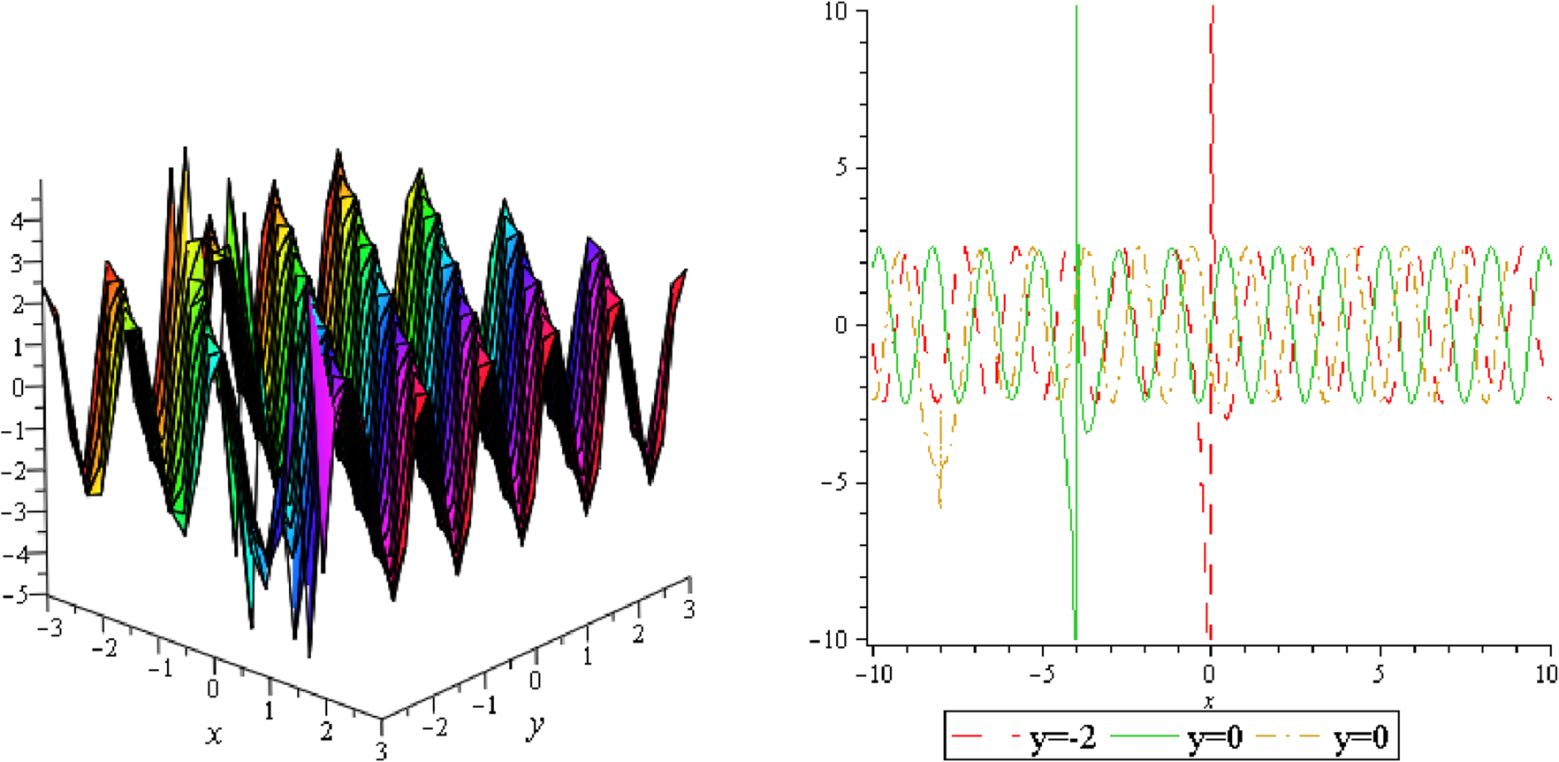
Periodic shape of $U_{3,4}$ for $a_{1} = 1$, $a_{2} = 2$, $a_{3} = 1$, $a_{4} = 4$, $k_{1} = 4$, $k_{5} = 5$, $h_{1} = \frac{1}{2}$, $h_{2} = 1$, $p = 1$, $q = 2$, $r = 1$, $\lambda = - 2$, $t = 0$ within the interval $- 3 \le x \le 3$ and $- 3 \le y \le 3$. The right figure displays the 2D plot, whereas the left figure displays the 3D plot for y = 2, y = 0 and y = − 2.

**Figure 14:**
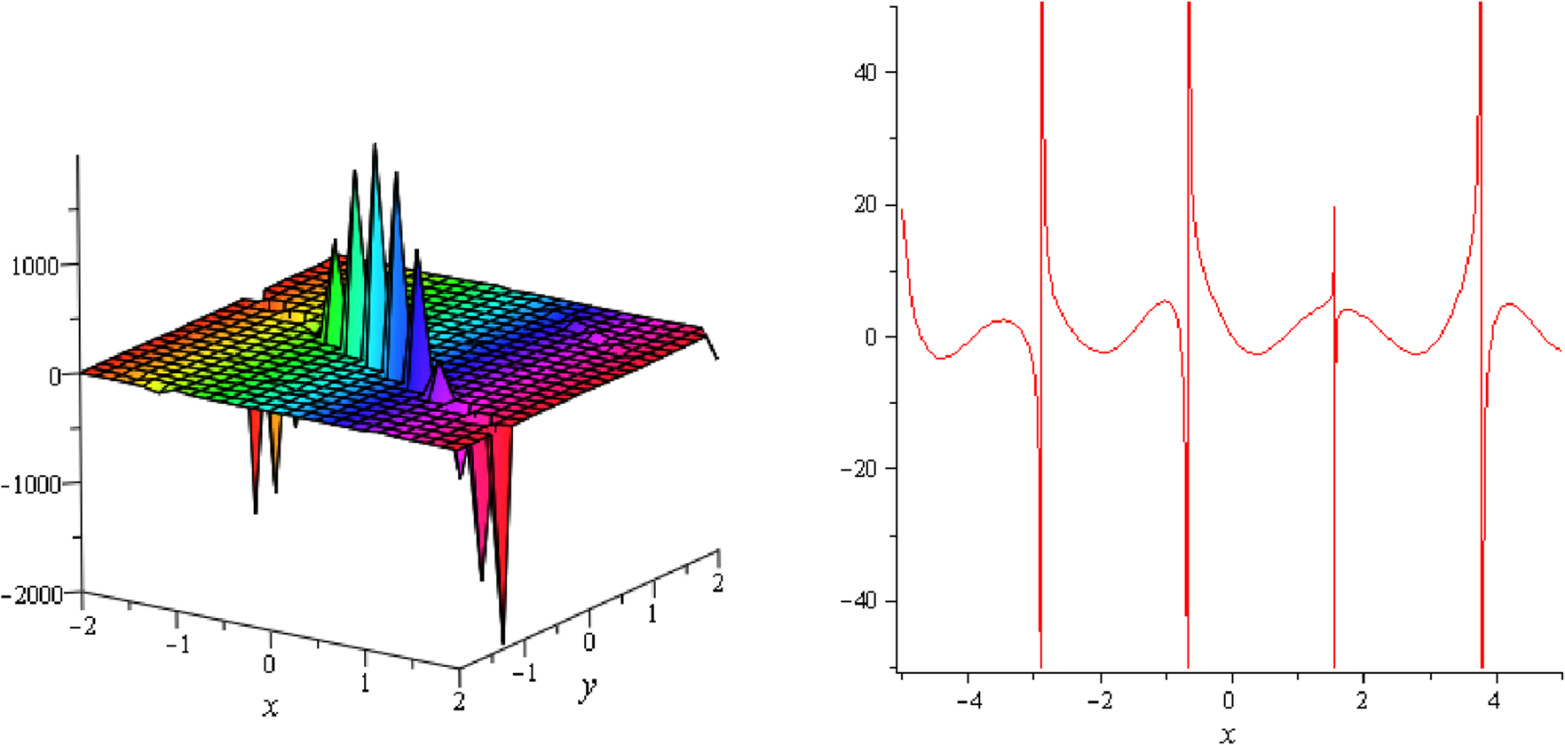
Periodic shape of $U_{3,5}$ for $a_{1} = 1$, $a_{2} = 2$, $a_{3} = 1$, $a_{4} = 4$, $k_{1} = 4$, $k_{5} = 5$, $h_{1} = \frac{1}{2}$, $h_{2} = 1$, $p = 1$, $q = 2$, $r = 1$, $\lambda = 2$, $t = 0$ within the interval $- 2 \le x \le 2$ and $- 2 \le y \le 2$. The right figure displays the 2D plot, whereas the left figure displays the 3D plot for y = 0. Which is the similar as the graph of $U_{3,6}$.

**Figure 15:**
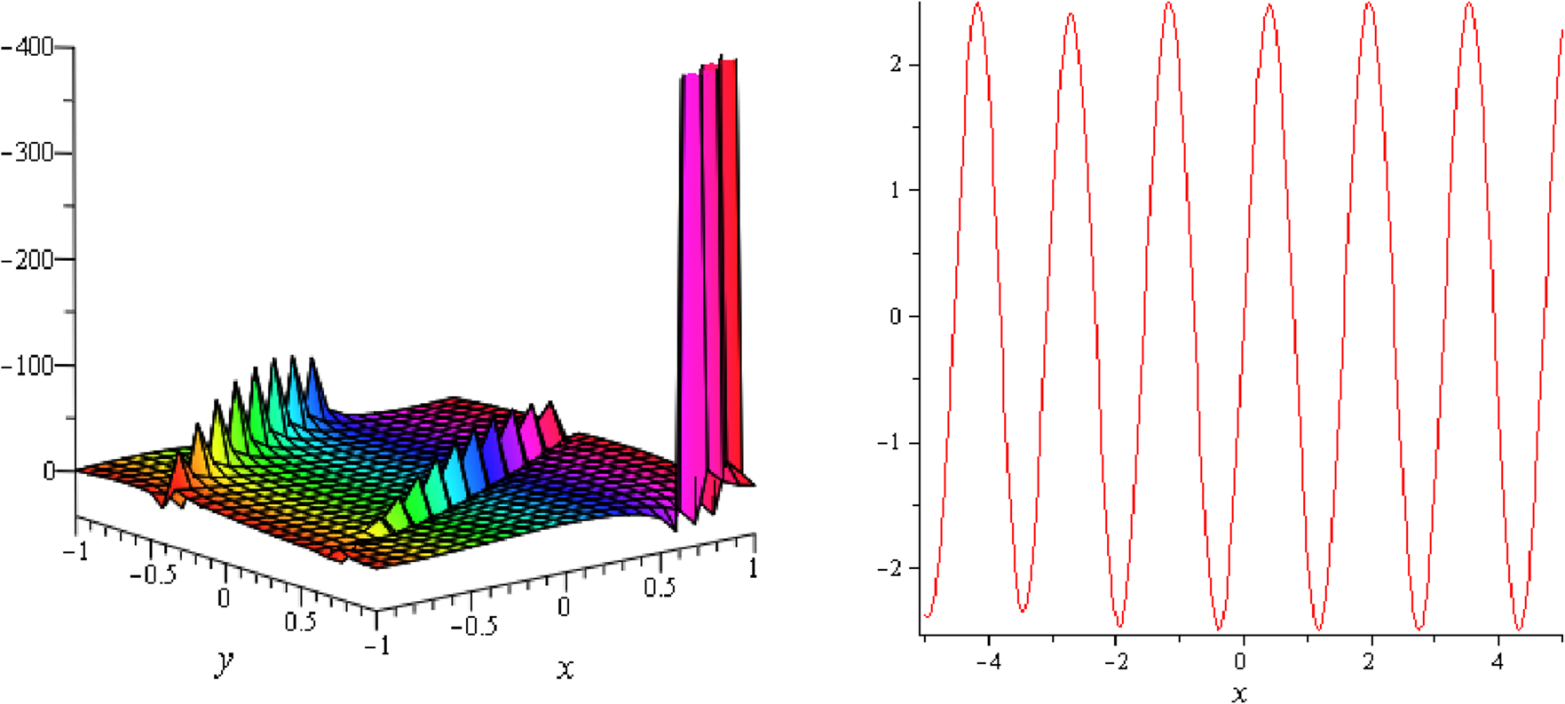
Periodic shape of $U_{3,7}$ for $a_{1} = 1$, $a_{2} = 2$, $a_{3} = 1$, $a_{4} = 4$, $k_{1} = 4$, $k_{5} = 5$, $h_{1} = \frac{1}{2}$, $h_{2} = 1$, $p = 1$, $q = 2$, $r = 1$, $\lambda = 2$, $t = 0$ within the interval $- 1 \le x \le 1$ and $- 1 \le y \le 1$.

## Discussion

The paper likely explores the existence and properties of optical solitons and rogue-type electrical solitons in the specific nonlinear equation describing the dynamics of spin waves in ferromagnetic materials. It might present analytical or numerical methods employed to find these soliton solutions, as well as their characteristics, stability regions, and possibly their interactions.

The novelty of the paper could lie in the application of soliton theory to the Heisenberg ferromagnetic spin chains equation on the unique behavior and properties of solitons in this particular physical system.

The unified method is used in this study to solve the nonlinear Schrödinger equation, which explains the nonlinear spin dynamics of the (2+1) dimensional Heisenberg ferromagnetic spin chains problem. Our attempts to solve these equations are successful. We give the parametric specifications necessary for a valid soliton to exist for each of the deduced solutions. We plot the 2D and 3D visuals in order to visualize some of the answers found. The investigation's reported findings may be helpful in clarifying the model's physical significance. These are a very important tool to analyze the propagation of electrical solitons, which propagate as voltage waves in nonlinear dispersive media, and to do other physical computations. The study's findings, which have been disclosed, might be useful in illuminating the models under consideration's physical significance and electrical field. We noticed that several of the solutions found in this study could be helpful in illuminating the physical significance of the investigated model as well as other nonlinear models in the nonlinear sciences.

It should be noticed that in all figures, the left side depicts 3D figure plotting and the right side depicts 2D figure plotting. Figure 1 is the similar as the graph of $U_{1,3}$ and $U_{1,4}$ within y = 2, y = 0 and y = − 2. Figure 2 describes the behavior as the graph of $U_{2,1}$, $U_{2,2}$,$U_{3,1}$ through y = 1, y = 0 and y = − 1. Figure 3 shows the 3D and 2D plotting for y = 1, y = 0 and y = − 1 and it is the similar as the graph of $U_{1,6}$. In Fig. 4, the periodic shape of $U_{1,7}$ shows the 3D and 2D plot for y = 1, y = 0 and y = − 1. Figure 5 displays the periodic shape of $U_{1,8}$ for y = 1 whilst Fig. 6 represents the periodic shape of $U_{1,7}$ within the interval $- 10 \le x \le 10$ and y = 1, y = 0 and y = − 1. Figure 7 shows periodic shape of $U_{2,3}$ at $t = 0$ within the interval $- 10 \le x \le 10$ and y = 1, y = 0 and y = − 1. Figure 8 indicates the periodic shape of $U_{2,3}$ within the interval $- 3 \le x \le 3$ and y = 1, y = 0 and y = − 1. In Fig. 9, the right figure displays the 2D plot, whereas the left figure displays the 3D plot for y = 1, y = 0 and y = − 1. which it is similar as the graph of $U_{2,6}$. Figure 10 describes the periodic shape of $U_{2,7}$ within the interval $- 8 \le x \le 8$ and $- 8 \le y \le 8$. Figure 11 is be periodic shape of $U_{2,9}$ within the interval $- 5 \le x \le 5$ and $- 5 \le y \le 5$ for y = 2, y = 0 and y = − 2. Figure 12 indicates the periodic shape of $U_{3,3}$ within the interval $- 3 \le x \le 3$ and $- 3 \le y \le 3$ for y = 0. Figure 13 depicts the periodic shape of $U_{3,4}$ within the interval $- 3 \le x \le 3$ and $- 3 \le y \le 3$ for y = 2, y = 0 and y = − 2 for both 2D and 3D plotting. Figure 14 represents the periodic shape of $U_{3,5}$ within the interval $- 2 \le x \le 2$ and $- 2 \le y \le 2$ for y = 0; Fig. 14 is the similar as the graph of $U_{3,6}$. Figure 15 is periodic shape of $U_{3,7}$ within the interval $- 1 \le x \le 1$ and $- 1 \le y \le 1$. The right figure displays the 2D plot, whereas the left figure displays the 3D plot for y = 1.

In the field of optical solitons, the trial solution you provided represents a possible form for the soliton profile. Solitons are self-reinforcing solitary waves that maintain their shape and propagate without dispersion. They are commonly observed in various nonlinear systems, including optical fibers.

In optical soliton systems, the equation you provided can be related to the nonlinear Schrödinger equation (NLSE) that describes the evolution of the soliton envelope. The terms involving k_1_, k_2_, h_1_, and h_2_ represent dispersion and nonlinearity coefficients, while the terms involving a_4_ represent the nonlinearity strength. The trial solution with different sets of l_0_, l_1_, and m_1_ corresponds to different types of soliton profiles that can emerge in the system. The constraints on l_0_, l_1_, and m_1_ in each set ensure the soliton solution satisfies certain properties or boundary conditions. The justification for the obtained constraint relations can be further explored by analyzing the dynamics of spin chains, which are relevant in various physical systems. In spin chain systems, the equation we provided can be related to the spin chain Hamiltonian and the corresponding equations of motion for the spin components. The trial solution with different sets of l_0_, l_1_, and m_1_ corresponds to different spin chain configurations and their dynamics. The constraints on l_0_, l_1_, and m_1_ in each set arise from the specific properties of the spin chain system, such as conservation laws, symmetries, or specific boundary conditions.

## Conclusion

The nonlinear spin dynamics of the (2+1)-dimensional Heisenberg ferromagnetic spin chains equation are described by a NLS type equation, which has been studied in this paper. We used a unified approach to derive generalized solutions for the (2+1) dimensional Heisenberg ferromagnetic spin chains equation's nonlinear spin dynamics in terms of hyperbolic and trigonometric function. We noticed that several of the solutions found in this study could be helpful in illuminating the physical significance of the investigated model as well as other nonlinear models in the nonlinear sciences.

## Data Availability

All data generated or analyzed during this study are included in this published article [and its supplementary information files including MAPLE software (Maplesoft)].
